# Development and validation of the Canine Reward Responsiveness Scale –Examining individual differences in reward responsiveness of the domestic dog

**DOI:** 10.1038/s41598-018-22605-1

**Published:** 2018-03-13

**Authors:** Linda Gerencsér, Nóra Bunford, Alexandra Moesta, Ádám Miklósi

**Affiliations:** 1 Eötvös Loránd University, Institute of Biology, Department of Ethology, 1117 Budapest, Pázmány Péter sétány 1/C, Hungary; 2 MTA-ELTE ‘Lendulet’ Neuroethology of Communication Research Group, Hungarian Academy of Sciences - Eotvos Lorand University, 1117 Budapest, Pázmány Péter sétány 1/C, Hungary; 3 Hungarian Academy of Sciences, Research Centre for the Natural Sciences, Institute of Cognitive Neuroscience and Psychology, 1117 Budapest, Magyar tudósok körútja 2, Hungary; 4 WALTHAM Centre for Pet Nutrition, Freeby Lane, Waltham-on-the-Wolds, Melton Mowbray, UK; 5 MTA-ELTE Comparative Ethology Research Group, Budapest, Hungary

## Abstract

Although there is ample data indicating that reward processing plays an important role in human psychopathologies and pharmaco- and psychotherapy treatment response, the corresponding animal-model research needs to be extended to models whose motivational and social dispositions are better generalizable than those of the traditional models. Accordingly, our aim was to develop and assess the reliability and validity of an owner-report rating scale of reward responsiveness in domestic dogs (*N* = 2149) and then to examine individual differences in reward responsiveness. Responsiveness was categorisable by reward type (ball/toy and food) and exhibited individual variability manifesting in age- and breed-related differences. Rating scale scores were associated with behavioural observation of reward processing, indicating evidence of convergent validity. Ball/toy and food reward responsiveness were associated with owner-rated hyperactivity-impulsivity‚ inattention and with differences in training, indicating evidence of concurrent validity. Extreme (vs. average) reward responsiveness was also predicted by dogs’ hyperactivity-impulsivity and inattention‚ and extreme responsiveness was associated with increased likelihood of physical health and/or social problems. These findings are informative with regard to the dog as an animal model for various human behavioural and cognitive functions‚ and also for the dog in its own right as they are relevant to training and welfare.

## Introduction

In accordance with the leading perspective put forth in the Research Domain Criteria (RDoC)^1^, reward processing is a multi-faceted construct comprised of approach motivation, initial and sustained responsiveness to reward attainment, reward learning and habit. The way in which individuals process and respond to rewarding stimuli (material, e.g., food, money or social, e.g., affiliative social contact) is crucial in a variable environment. Individual differences in reward processing are associated both with psychiatric disorders, such as behavioural, neurodevelopmental and substance use disorders‚ and with transdiagnostic characteristics of these disorders, such as emotion dysregulation (ED). The relevant behavioural, neurodevelopmental and substance use disorders include Oppositional Defiant (ODD) and Conduct (CD) Disorders, Attention Deficit/Hyperactivity (ADHD) and Autism Spectrum (ASD) Disorders, psychoactive substance and behavioural/nonsubstance addictions^2–7^. These disorders are common^8–10^, impairing, and costly. Impairments include academic problems and social difficulties in the home, peer, and school settings^11,12^. Costs include personal and financial costs to affected youth, families, and society^13^. Impairments and costs are exacerbated by correlates of reward processing that may occur independent of these disorders, such as ED^12^.

Directives have been increasingly focused on adoption of a biologically-based and informed approach to the conceptualization, research and treatment of psychiatric disorders (e.g., RDoC), with reward processing being one of the most well-studied neurobiological mechanisms in the human literature. Advances in research on humans, however, have not been paralleled by corresponding research on animal models, despite importance of, and utility in, validating and working with such models^14–16^. Although there is ample research on reward processing and its abnormalities in rodents^17–23^, with results generally indicating similar neural mechanisms underlying these phenomena as in humans^24,25^, there are limitations to the rodent model. These limitations include, for example, a restricted laboratory environment as living space in comparison to the complex and variable environment of human societies and the need for fluid and/or food restriction as a motivational tool – all of which can considerably impact the degree to which rodent data can be generalized to humans.

An emerging and promising model of human behaviour is the domestic dog (*Canis familiaris*). Advantages of the dog lie, in part, in dogs exhibiting a range of socio-cognitive skills that share key behavioural and functional characteristics with those of humans^26^ and that they also physically and socially share the environment with humans. Importantly‚ with regard to reward processing, dogs’ natural cooperativeness and trainability obviate the need for fluid and/or food restriction. As such, relative to other species, when a dog participates in an experiment, its physiological and social state is more similar to that of humans’^15^. Indeed, psychometric rating scales originally designed to measure human behaviour have been successfully adapted as human-report measures of dog behaviour, similar to parent-report measures of child functioning^27–29^. It is of no surprise, given these considerations, that over the last 20 years, canine ethological research^30^ has focused on the dog as a model for human behaviours and neurodevelopmental^31^ and psychiatric disorders, including ones relevant to reward processing, such as inequity aversion^32^, inhibition^33,34^, social cause and effect (contingency) detection^35^, social learning^36^ as well as ADHD^27,37^.

Further underscoring the importance of reward processing in both humans and dogs is that individual differences in reward processing have implications for pharmaco- and psychotherapy response (in case of humans) and training (in case of dogs), which, in turn, are relevant to improving the functioning of youth and adults with symptoms of neurodevelopmental or psychiatric disorders and to animal welfare, respectively. Among the non-pharmacological, i.e. behavioural or psychosocial interventions with the most empirical evidence for treating children with ODD, CD, and ADHD are behavioural parent training, behavioural classroom management and behavioural peer interventions^38,39^. Basic principles of behaviour management‚ that is, reinforcement and punishment, are at the core of these interventions^38,39^. As such, it stands to reason that individual differences in reinforcement sensitivity, i.e., reward sensitivity and processing are associated with differential treatment response.

Regarding dogs, recently, there has been a marked change in dog training. As opposed to punishment-based methods, reward-based ones, such as using food, toys, play and other forms of social interaction as positive reinforcement have gained considerable support from experts^40^. One of the main causes of this shift are data indicating that dogs who are trained primarily with reward-based methods have better welfare – i.e. exhibit a lower number of problematic behaviours – as compared to dogs who are punished more frequently^41^. In addition, owners also report an overall lower number of undesirable behaviours in dogs trained without the use of punishment-based techniques^42^. In some aspects, dog training can be regarded as similar to behavioural interventions for affected children, since it uses the same basic principle(s) of reinforcement (and punishment). Thus, individual differences in dogs in reward sensitivity may have implications for both short and long-term response to training and for other aspects of welfare.

Considering the above, it is surprising that – to the authors’ knowledge – there is no research focusing on individual variation in dogs’ sensitivity to rewards (i.e., food and ball/toy rewards, which are most often used during training), and although a psychometric scale measuring a construct relevant to reward responsiveness, i.e. negative or positive activation tendencies/emotional predispositions^43^ is available, there is no reliable, validated measure of reward responsiveness *per se* in dogs. Accordingly, our aim in this questionnaire study supported by behavioural measures was to develop and validate an owner-report rating scale measure of reward (i.e., food and ball/toy) responsiveness in dogs, i.e., the Canine Reward Responsiveness Scale (CRRS), as well as to characterize a sample of dogs using that rating scale, with regard to aspects of reward responsiveness. We hypothesized that (1) there is individual variation in dogs’ responsiveness to food and ball/toy rewards as reflected by both the rating scale and by pertinent behavioural correlates measured via behavioural observation in a controlled laboratory setting. We further expected that (2) variation in reward responsiveness is related to the type of reward used during training and other variables related to training, measured via owner-reported reward frequency and increase in the dog’s motivation over the course of training, and that (3) excessive reward pursuing is associated with other, relevant behavioural traits, such as the dog’s owner-reported attachment to favourite objects/toys; inattention, hyperactivity-impulsivity on a psychometrically validated rating scale; and with owner-reported indices of welfare, i.e., problems with physical health and social functioning.

## Results

### Confirmatory Factor Analyses (CFA)

Of all 30 corresponding items of our developed questionnaire (15 referring to ball/toy and 15 to food reward responsiveness, see Methods and Supplementary Text S1), considering modification and model fit indices as well as standardized factor loadings, 14 ball/toy reward responsiveness questions were retained, and these loaded onto one factor (f1, Ball/toy responsiveness, B/TR, *M* = 2.91, *SD* = 0.95) and 10 food reward responsiveness questions were retained, and these loaded onto a second factor (f2, Food responsiveness, FR, *M* = 3.00, *SD* = 0.91) (see Table 1). Final model fit was approaching, but not conformably achieving excellent levels across fit indices, χ^2^(225) = 1418.52, *p* < 0.001; RMSEA = 0.050 (95% CI: 0.047, 0.052); CFI = 0.94. All items had a standardized factor loading estimate of ≥0.40, further indicating sufficient fit. Both factors demonstrated acceptable internal consistency (B/TR α = 0.897, FR α = 0.846), while the correlation between the factors was negligible (*r* = −0.003).

**Table 1:** CRRS items loading on one of the two factors as identified by the CFA.

Factor 1	Factor 2
(b/t1) Is pushy when wants to play with ball/toy	(f1) Is pushy when wants to get food
(b/t2) Initiates play with ball/toy even with unfamiliar people	(f2) Goes to unfamiliar people to beg for food
(b/t3) Runs eagerly after the thrown ball/object	(f3) Wolfs down the food
(b/t4) Is responsive if cannot play balls/play with other objects at the usual place and/or time	(f4) Is responsive if does not receive food at the usual time
(b/t5) Initiates play with ball/other objects	
(b/t6) Is so focused on playing with ball/objects that hardly notices what is happening around	(f6) Is so focused on eating that hardly notices what is happening around
(b/t7) Gets excited if he/she can play balls/fetch objects	(f7) Gets excited if he/she can get food
(b/t8) Quits playing with ball/other objects on his/her own (R) ^a^	(f8) Leaves leftover food after the usual feeding (R) ^a^
(b/t9) Can be motivated by ball/toy to do/tolerate things he/she does not like otherwise	
(b/t10) Is tireless with playing with ball/objects	(f10) Has a voracious appetite
(b/t11) Is easily distracted from playing ball/fetching objects (R) ^a^	
(b/t12) Does not indicate that he/she would like to play with ball/toys (R) ^a^	
(b/t13) Only plays ball/fetches objects when is in a playful mood (R) ^a^	(f13) Only shows interest in the food when is really hungry (R) ^a^
(b/t14) Readily plays with any object	(f14) Readily eats anything
Interpretation	
Ball/toy responsiveness (B/TR, α = 0.897)	Food responsiveness (FR, α = 0.846)

Note. R = reversed item. All retained items had a standardized factor loading estimate ≥0.40.

### Evidence of Validity across Factors

Assumptions of statistical tests were considered prior to (and following, where appropriate) analyses; these were satisfied. For conceptualization and definition of variables of interest, see Methods and Supplementary Tables S2 and S5.

#### Evidence of convergent validity – Relationship to independent items and behavioural measures

Independent (of the CRRS) questionnaire items reflecting the owners’ opinions about dog behaviour reflective of ball/toy and food reward pursuing in general (see Text S1) were positively associated with the corresponding CRRS factors (*r* = 0.767, *p* < 0.001 and *r* = 0.705, *p* < 0.001, respectively).

Associations between observable behaviour in laboratory tests that we carried out on a subsample of dogs in the context of two reward responsiveness paradigms (involving attainable and unattainable food or ball/toy rewards, see also Methods and Text S3) and the subjects’ corresponding CRSS factors are summarized in Table 2. Positive associations were observed between B/TR and time spent near both the attainable (*r* = 0.374, *p* = 0.021) and unattainable ball/toy reward (*r* = 0.602, *p* < 0.001), manipulating both the reward (*r* = 0.413, *p* = 0.012) and the apparatus to obtain the unattainable reward (*r* = 0.393, *p* = 0.016), focusing on the unattainable reward (*r* = 0.694, *p* < 0.001) and the frequency of returning to the unattainable reward (*r* = 0.546, *p* = 0.001). B/TR negatively correlated with latency of approaching the unattainable reward (*r* = −428, *p* = 0.009). FR positively correlated with frequency of returning to the location of reward consumption (*r* = 0.495, *p* = 0.003), focusing on the unattainable reward (*r* = 0.562, *p* = 0.001) and manipulating the apparatus to obtain the unattainable reward (*r* = 0.392, *p* = 0.016)‚ and FR negatively correlated with latency of approaching the attainable food reward (*r* = −410, *p* = 0.012).

**Table 2:** Associations between behavioural measures and the corresponding CRSS factors.

	Behavioural paradigm	Short description of measured behaviour	Correlation (Pearson’s *r*) with FR score^a^
Food responsiveness	Food reward attainable	Latency to approach and eat the food (*s*)	−0.410 (*p* = 0.012)^*^
		Time spent near location of food consumption (%)	0.198 (*p* = 0.147)
		Time spent manipulating the apparatus at location of food consumption (%)	0.210 (*p* = 0.133)
		Return frequency to location of food consumption (*n*)	0.495 (*p* = 0.003)^*^
	Food reward unattainable	Latency to approach the food containing apparatus (*s*)	0.155 (*p* = 0.206)
		Time spent focusing on the unattainable food (%)	0.562 (*p* = 0.001)^*^
		Time spent manipulating the food containing apparatus (%)	0.392 (*p* = 0.016)^*^
		Time spent near the food containing apparatus (%)	0.237 (*p* = 0.104)
		Return frequency to the food containing apparatus (*n*)	0.261 (*p* = 0.082)
			Correlation (Pearson’s *r*) with B/TR score^a^
Ball/toy responsiveness	Ball/toy reward attainable	Latency to approach and touch the ball/toy (*s*)	0.086 (*p* = 0.325)
		Time spent near the ball/toy (%)	0.374 (*p* = 0.021)^*^
		Time spent manipulating the ball/toy (%)	0.413 (*p* = 0.012)^*^
		Return frequency to the ball/toy (*n*)	0.281 (*p* = 0.066)
	Ball/toy reward unattainable	Latency to approach the ball/toy containing apparatus (*s*)	−0.428 (*p* = 0.009)^*^
		Time spent focusing on the unattainable ball/toy (%)	0.694 (*p* = 10^–5^)^*^
		Time spent manipulating the ball/toy containing apparatus (%)	0.393 (*p* = 0.016)^*^
		Time spent near the ball/toy containing apparatus (%)	0.602 (*p* = 0.000215)^*^
		Return frequency to the ball/toy containing apparatus (*n*)	0.546 (*p* = 0.001)^*^

Note. ^a^*n* = 30. ^*^Significant result (following Benjamini-Hochberg correction for multiple comparisons).

#### Evidence of concurrent validity – Relationship to conceptually related phenomena

There were main effects of age *F*(2, 2054) = 9.455, *p* < 0.004; Pillai’s trace (V) = 0.009; breed group *F*(22, 4110) = 6.006, *p* < 0.001; Pillai’s V = 0.062; toy/object attachment *F*(6, 4110) = 46.27, *p* < 0.001; Pillai’s V = 0.127; and the second hyperactivity-impulsivity subscale (HY2; with higher scores reflecting greater difficulties with hyperactivity-impulsivity, derived from a validated, independent owner-report rating scale of dog attention and activity/impulsivity – for more detail see the Methods) *F*(2, 2054) = 108.81, *p* < 0.001; Pillai’s V = 0.096. Age was negatively associated with B/TR and positively with FR. Basal breed dogs were rated lowest and lower than almost all other groups on B/TR, while herding dogs and retrievers were rated the highest, and higher than the basal, toy, unclassified purebred and cross-bred groups. Retrievers were rated the highest, higher than all other groups on FR (see Supplementary Table S2). Ball/toy responsiveness was different across all levels of toy/object attachment, exhibiting an increase with increasing attachment (see Supplementary Table S2). HY2 exhibited a positive linear relationship with both factors.

**Figure 1:**
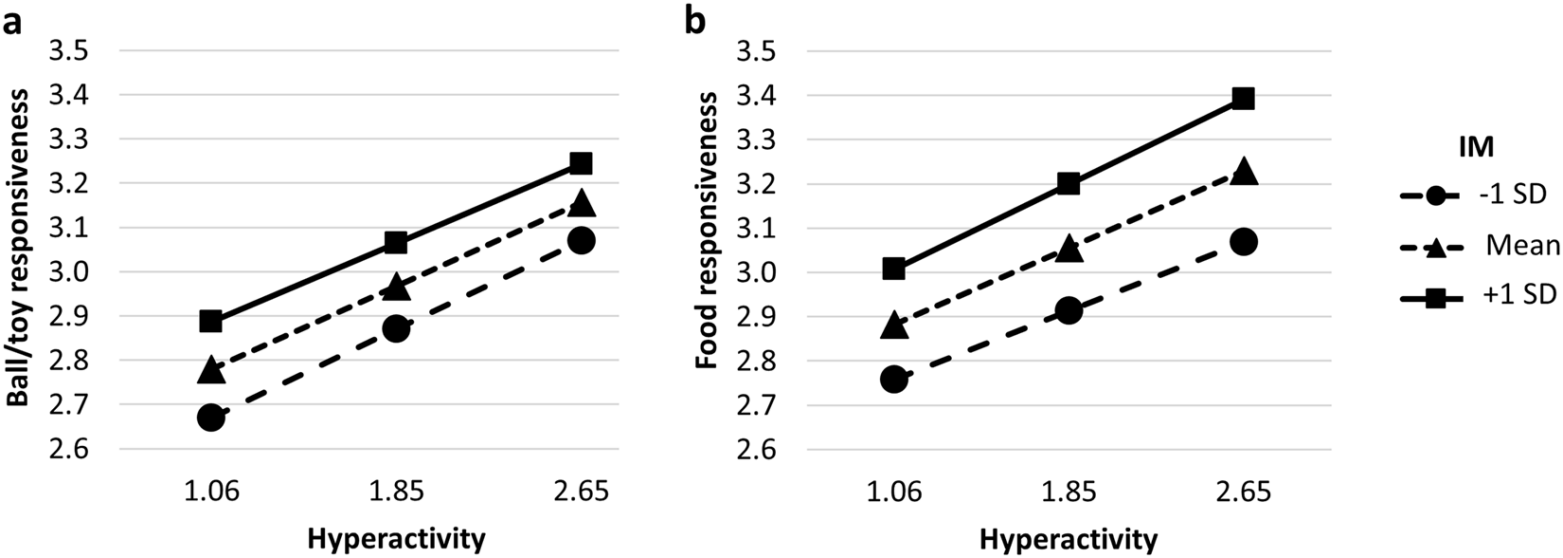
Estimated Marginal Means of Ball/toy (**a**) and Food (**b**) responsiveness and Hyperactivity at Levels of Increase in motivation (IM). (**a**) Greater Hyperactivity in combination with greater Ball/toy responsiveness are associated with greater Increases in motivation. (**b**) Greater Hyperactivity in combination with greater Food responsiveness are not associated with greater Increases in motivation. The different lines stand for the three different levels of IM; dashed with triangles: mean, solid with rectangles: mean + 1 SD, dashed with circles: mean − 1 SD.

There was an interaction effect between Increase in motivation (i.e., the degree to which, over time, it became easier to motivate the dog with reward during training) and the first hyperactivity-impulsivity subscale (HY1; with higher scores reflecting greater difficulties with hyperactivity-impulsivity) on B/TR *F*(2, 2054) = 4.064, *p* = 0.017; Pillai’s trace (V) = 0.004, where at higher levels of Increase in motivation, greater HY1 corresponded to greater B/TR (Fig. 1). There was no interaction effect between Increase in motivation and HY1 on FR (*p* = 0.073) (Fig. 1).

**Figure 2:**
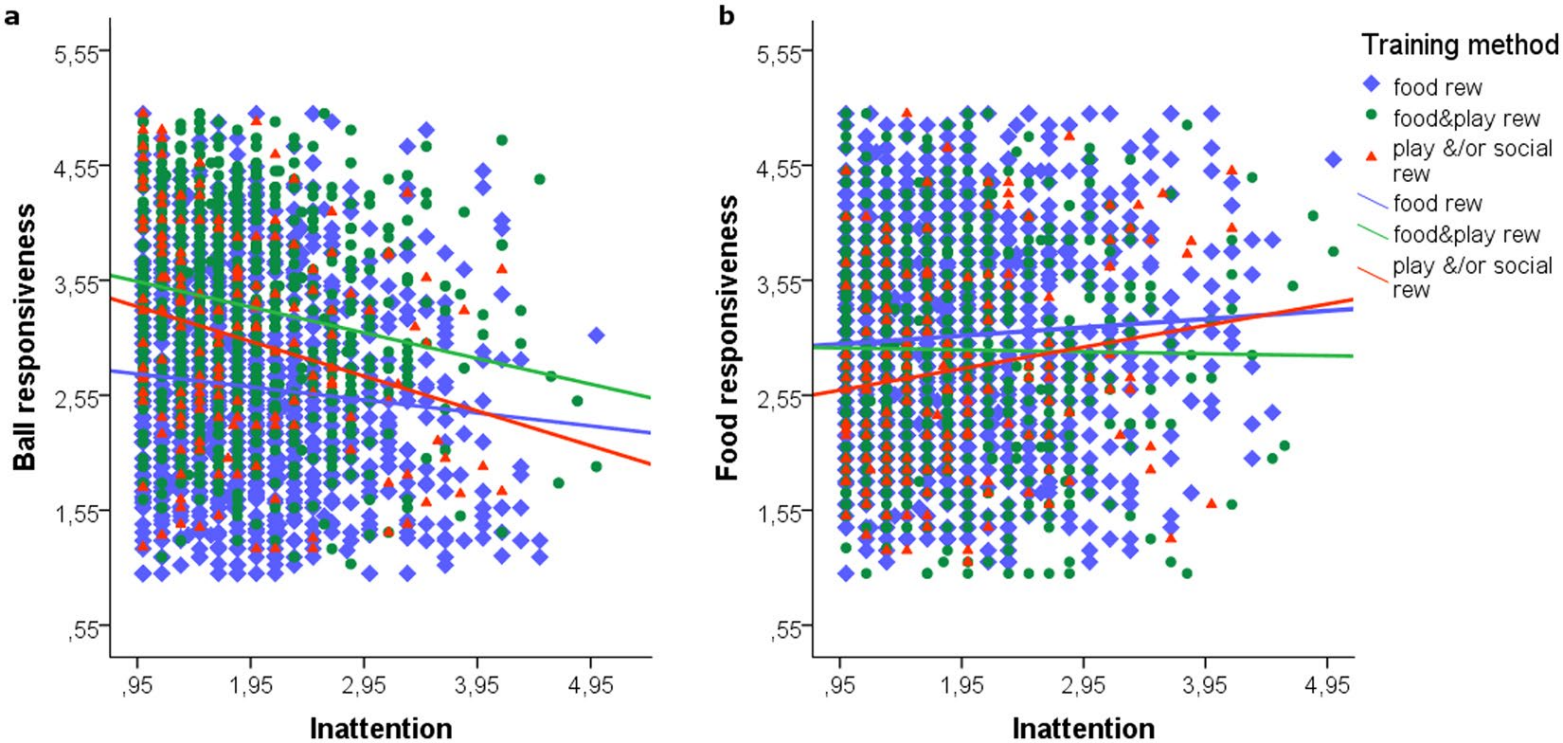
Estimated Marginal Means of Ball/toy (**a**) and Food (**b**) responsiveness at levels of Training method. (**a**) A negative relationship between Inattention and Ball/toy responsiveness was seen in all dogs but the strength of this relationship varied depending on Training method. The strongest negative association between Inattention and Ball/toy responsiveness was observed in dogs who were rewarded by ball/toy or social reinforcement (as shown in red – ‘play &/or social rew’), then in dogs rewarded by food and ball/toy (as shown in green – ‘food & play rew’), and the weakest in dogs rewarded by food only (as shown in blue – ‘food rew’). (**b**) There were no differences in the direction or magnitude of association between Inattention and Food responsiveness given the type of reward dogs received during training (i.e. training method). As for training method types, ball/toy or social reinforcement is shown in red (play &/or social rew), reinforcement by food and ball/toy is shown in green (food & play rew), and reinforcement by food reward only is shown in blue (food rew).

There was also an interaction effect between Training method (i.e., the method of reward predominantly used during the dog’s training) and Inattention (IA; with higher scores reflecting greater difficulties with inattention) on B/TR (Fig. 2) *F*(4, 4110) = 3.242, *p* = 0.012; Pillai’s trace (V) = 0.006, where the strongest negative association between IA and B/TR was observed in dogs who were rewarded by play (ball/toy) or social reinforcement, then in dogs rewarded by food and play (ball/toy), and the weakest in dogs rewarded by food only.

**Figure 3:**
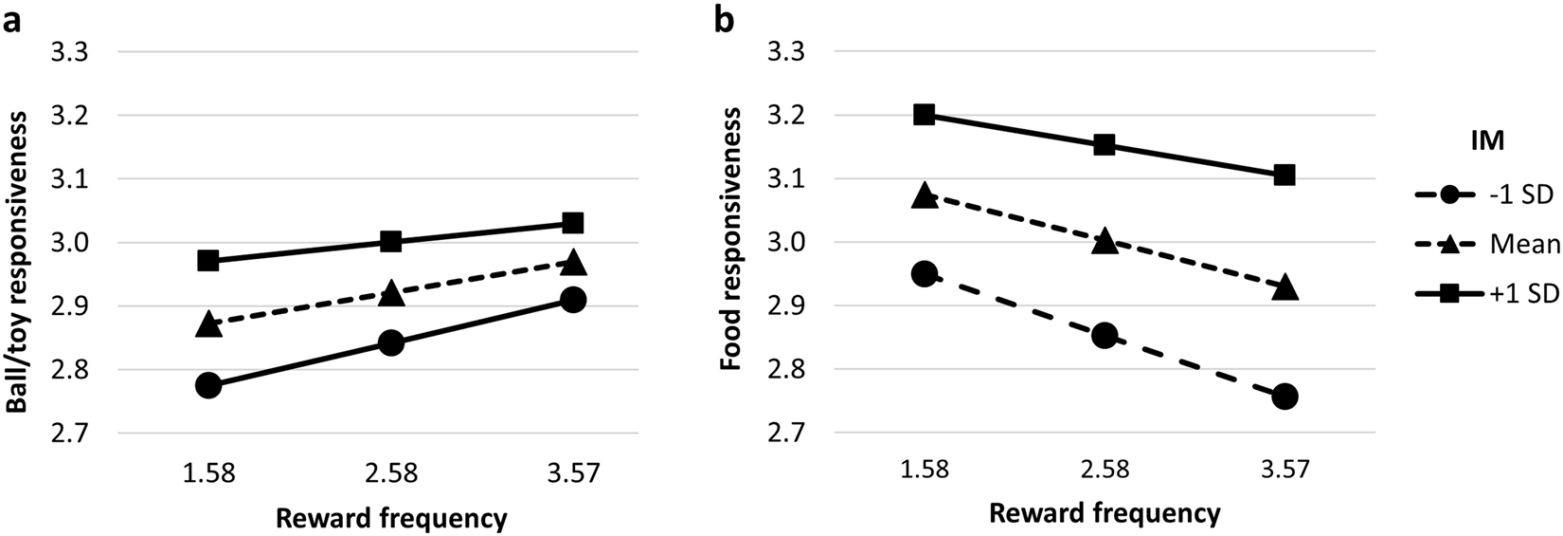
Estimated Marginal Means of Ball/toy (**a**) and Food (**b**) responsiveness and Reward frequency at Levels of Increase in motivation (IM). (**a**) At increasing levels of IM, greater Reward frequency (RF) corresponded to greater Ball/toy responsiveness. (**b**) At increasing levels of IM, greater RF contributed to lower Food responsiveness. The different lines stand for the three different levels of IM; dashed with triangles: mean, solid with rectangles: mean + 1 SD, dashed with circles: mean – 1 SD.

Finally, there was an interaction effect between Increase in motivation and Reward frequency on both B/TR and FR (Fig. 3) *F*(6, 4110) = 3.121, *p* = 0.005; Pillai’s trace (V) = 0.009, where at increasing levels of Increase in motivation, greater Reward frequency corresponded to greater B/TR but at increasing levels of Increase in motivation, greater Reward frequency contributed to lower FR.

#### Evidence of concurrent validity – Predicting extreme group membership given conceptually related phenomena

The overall model (i.e., predicting membership in either an extreme low or high ball/toy responsive or an extreme low or high food responsive group or in combination of extreme groups, relative to an average group, from IA, HY1, HY2, Traning method, Reward frequency, and Increase in motivation) fit the data well (Pearson *χ*^2^(1204) = 954.230, *p* = 1.000) and predicted group membership (*p* < 0.001), accounting for 80.4% (Nagelkerke *R*^2^) of the variance in such membership (for the formation of groups see the Methods/Analytic plan section).

It was more likely for dogs to be in the Low toy/ball and food responsive group (LTFR) than in the Average toy/ball and food responsive group (ATFR), if the frequency with which they were rewarded interacted with their HY2, IA, and HY1 scores. Among those in the LTFR group, dogs who were rewarded *monthly* as opposed to *daily more times* were more likely to have higher HY2 (WALD = 4.344, *df* = 1, *p* = 0.037, Exp(B) = 0.022 [95%CI = 0.001; 0.796) and IA scores (WALD = 4.529, *df* = 1, *p* = 0.033, Exp(B) = 0.033 [95%CI = 0.001; 0.765) whereas there were no differences in HY2 or IA among dogs who were rewarded *weekly* vs. *daily more times* or *daily once* vs. *daily more times*. Both dogs who were rewarded *monthly* as opposed to *daily more times* (WALD = 7.965, *df* = 1, *p* = 0.005, Exp(B) = 4787.245 [95% CI = 13.315;1721259.101) and dogs who were rewarded *weekly* vs. *daily more times* (WALD = 4.441, *df* = 1, *p* = 0.035, Exp(B) = 147.648 [95% CI = 1.418; 15272.605) were more likely to have higher HY1 scores.

It was more likely for dogs to be in the High toy/ball responsive group (HTR) than in the average group, if the frequency with which they were rewarded interacted with their HY2 and HY1 scores. Higher HY2 scores were more likely both in dogs who were rewarded *monthly* as opposed to *daily more times* (WALD = 7.010, *df* = 1, *p* = 0.008, Exp(B) = 0.001 [95%CI = 7.013^e−6^;0.170) and in dogs who were rewarded *weekly* vs. *daily more times* (WALD = 3.876, *df* = 1, *p* = 0.049, Exp(B) = 0.001 [95% CI = 0.000; 0.982). Higher HY1 scores were more likely in dogs who were rewarded *monthly* as opposed to *daily more times* (WALD = 7.134, *df* = 1, *p* = 0.008, Exp(B) = 8257.427 [95%CI = 11.030; 6181590.891).

It was more likely for dogs to be in the High toy/ball and food responsive group (HTFR) than in the average group, if they had higher HY1 scores (WALD = 32.420, *df* = 1, *p* < 0.001, Exp(B) = 3.071^e−14^ [95% CI = 6.854^e−19^; 1.376^e–9^); if there was a positive association between their IA and HY1 scores (WALD = 6.307, *df* = 1, *p* = 0.012, Exp(B) = 0.108 [95% CI = 0.019; 0.614); and if the method with which they were trained interacted with their HY1 scores. Dogs who were trained using food reward had higher HY1 scores than dogs who were trained using play/social reward (WALD = 861.503, *df* = 1, *p* < 0.001, Exp(B) = 9.342^E+15^ [95% CI = 8.017^e+14^; 1.089^e+17^) but there was no difference in HY1 scores between dogs who were trained with food and play/social reward and dogs who were trained using play/social reward.

#### Evidence of concurrent validity – Extreme group membership predicting outcomes of physical health and social functioning

**Figure 4:**
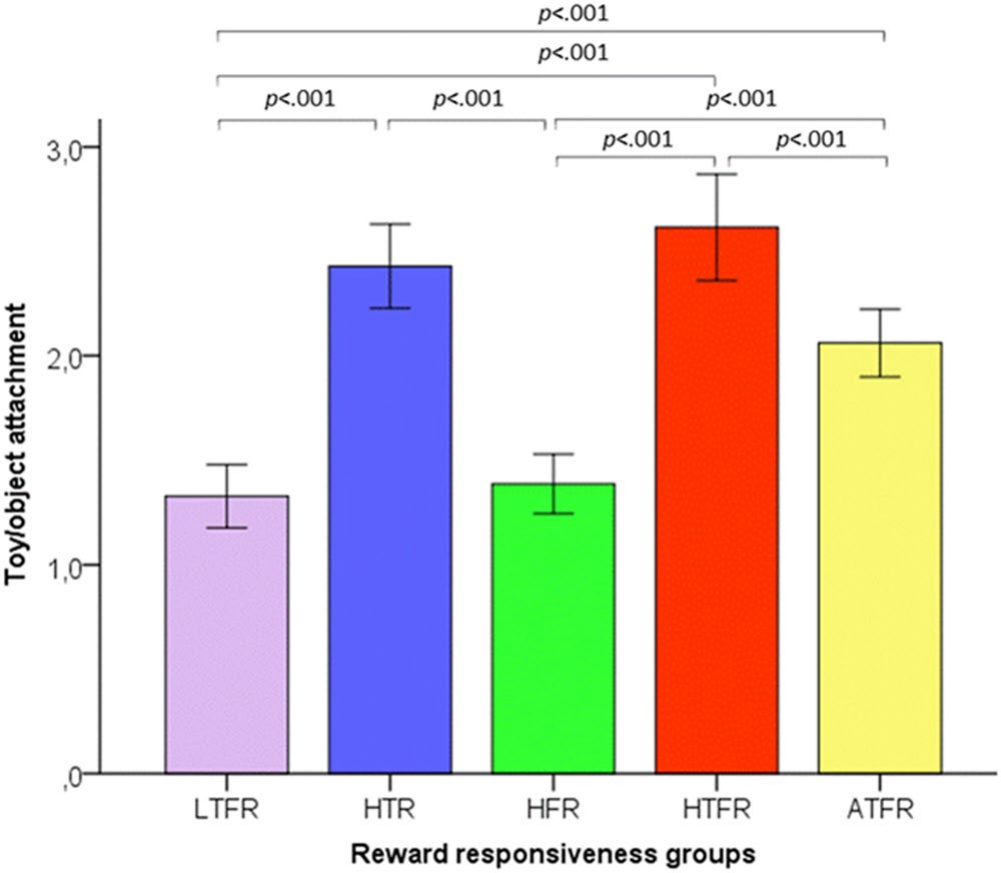
Differences in Toy/object attachment between dogs average and extreme on Ball/toy and/or Food responsiveness. Dogs high on Ball/toy and low on Food responsiveness (HTR) had higher scores than dogs in the other groups, except for dogs high on both Ball/toy and Food responsiveness (HTFR) or dogs average on Ball/toy and Food responsiveness (ATFR, i.e., average on reward responsiveness) on Toy/object attachment. Dogs high on both Ball/toy and Food responsiveness (HTFR) scored higher than dogs average on reward responsiveness (ATFR). The error bars represent +/− 2 SE.

**Figure 5:**
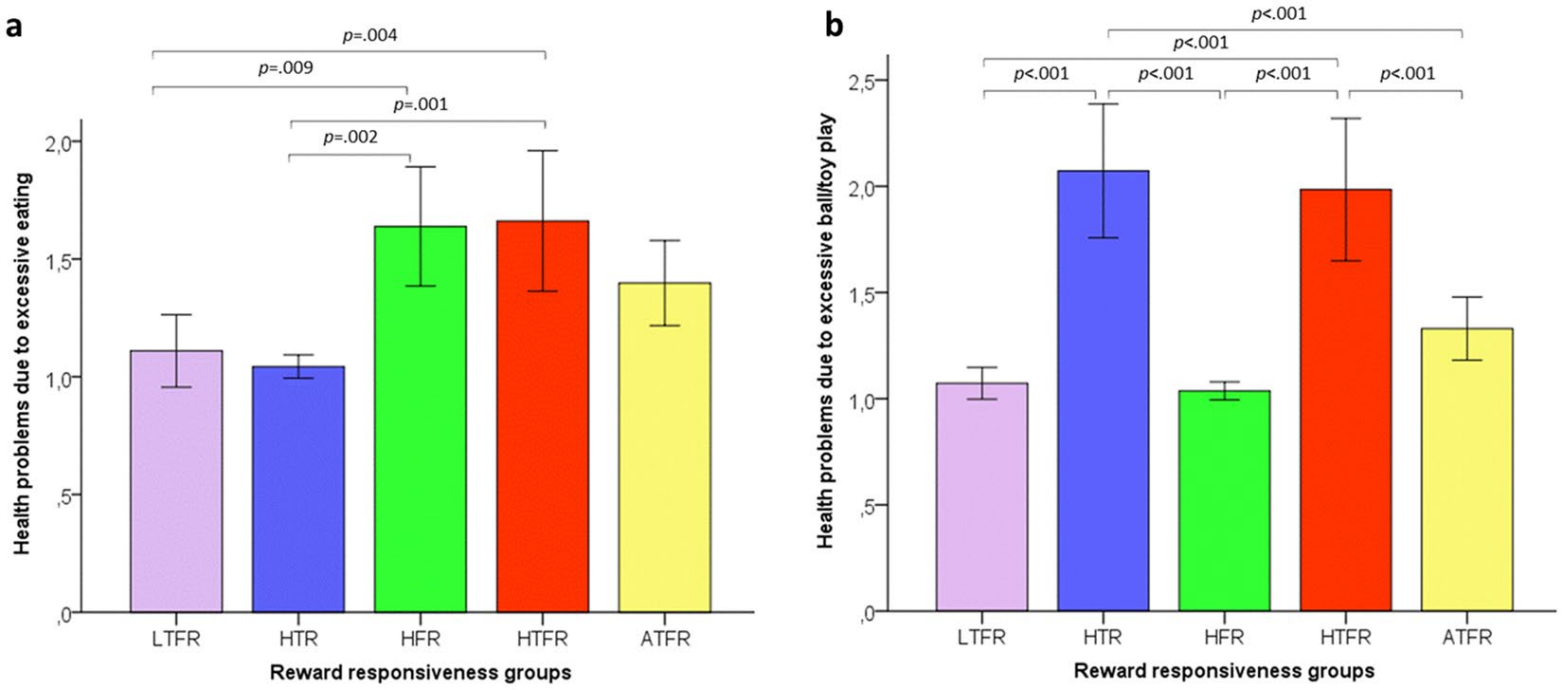
Differences in health problems due to excessive food (**a**) and ball/toy (**b**) reward pursuing between dogs average and extreme on Ball/toy and/or Food responsiveness. (**a**) Dogs low on both Ball/toy and Food responsiveness (LTFR) were rated lower than dogs low on Ball/toy and high on Food responsiveness (HFR) and dogs high on both Ball/toy and Food responsiveness (HTFR) on health problems due to excessive food reward pursuing. The HFR group was rated as having more eating-related health problems than dogs high on Ball/toy and low on Food responsiveness (HTR), while there was no difference between dogs low on Ball/toy and high on Food responsiveness (HFR) and dogs high on both Ball/toy and Food responsiveness (HTFR) or dogs average on reward responsiveness (ATFR) on health problems due to excessive food reward pursuing. (**b**) Dogs high on Ball/toy and low on Food responsiveness (HTR) were rated higher than dogs low on Ball/toy and high on Food responsiveness (HFR), HTR dogs did not differ from dogs high on both Ball/toy and Food responsiveness (HTFR), while the latter were rated higher than dogs average on reward responsiveness (ATFR) on health problems due to excessive ball/toy reward pursuing. The error bars represent +/−2 SE.

**Figure 6:**
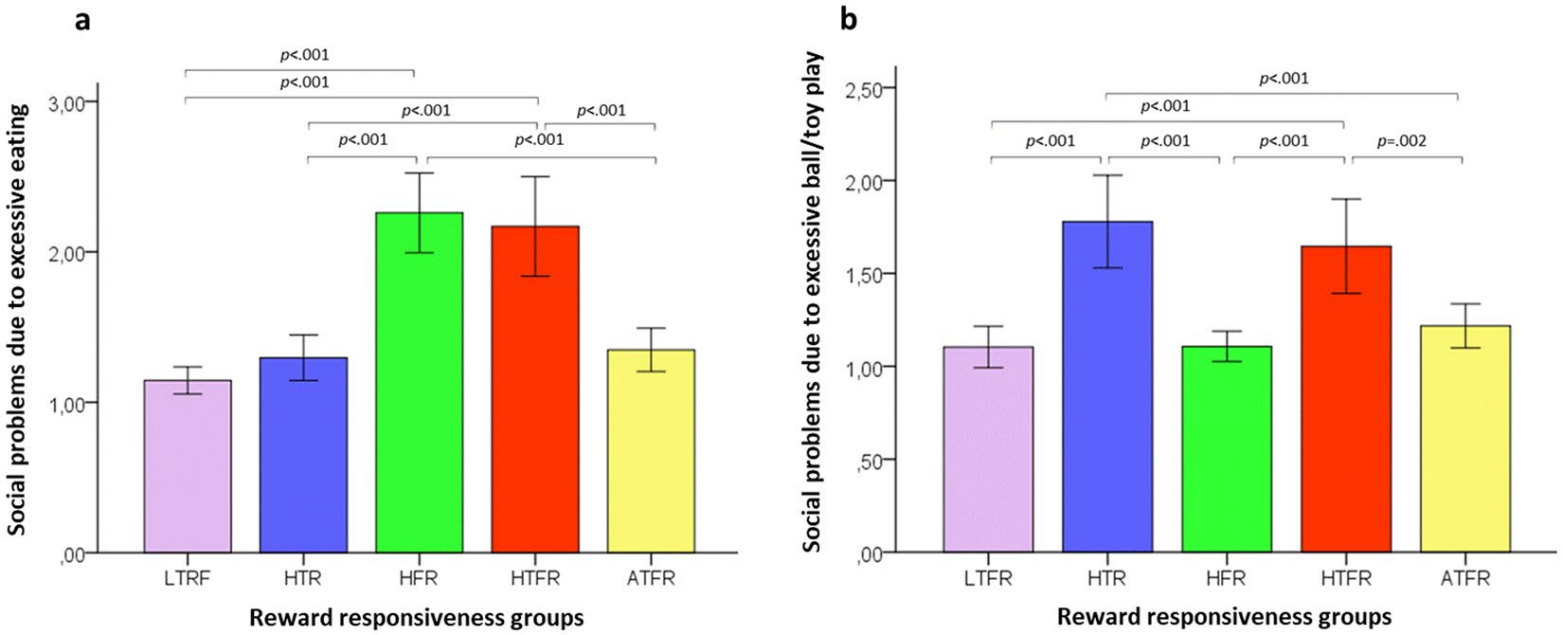
Differences in social problems due to excessive food (**a**) and ball/toy (**b**) reward pursuing between dogs average and extreme on Food and/or Ball/toy responsiveness. (**a**) Dogs high on Ball/toy and low on Food responsiveness (HTR) were rated lower than both dogs low on Ball/toy and high on Food responsiveness (HFR) and dogs high on both Ball/toy and Food responsiveness (HTFR) on social problems due to excessive food reward pursuing. The HFR group did not differ from the HTFR group but was rated higher than dogs average on reward responsiveness (ATFR), and the HTFR group was rated also higher than the average group on social problems due to excessive food reward pursuing. (**b**) Dogs high on Ball/toy and low on Food responsiveness (HTR) received higher scores than both dogs low on Ball/toy and high on Food responsiveness (HFR) and dogs average on reward responsiveness (ATFR) but did not differ from dogs high on both Ball/toy and Food responsiveness (HTFR). The HTFR group were rated higher than average dogs on social problems due to excessive ball/toy reward pursuing. The error bars represent +/−2 SE.

A test of the omnibus model indicated differences among the above mentioned groups in outcome variables of interest, *F*(20,1492) = 14.349, *p* < 0.001, *η*_*p*_^2^ = 0.161). Specifically, there were group differences in toy/object attachment, *F*(4,1492) = 36.633, *p* < 0.001, *η*_*p*_^2^ = 0.282, problems with physical health due to excessive eating, *F*(4,1492) = 6.768, *p* < 0.001, *η*_*p*_^2^ = 0.067 and due to excessive playing with ball/toys, *F*(4,1492) = 22.660, *p* < 0.001, *η*_*p*_^2^ = 0.195, problems with social functioning due to excessive eating, *F*(4,1492) = 23.790, *p* < 0.001, *η*_*p*_^2^ = 0.203 and excessive playing with ball/toys *F*(4,1492) = 13.267, *p* < 0.001, *η*_*p*_^2^ = 0.124). All results are depicted in Figs 4–6; key findings are summarized below.

With regard to toy/object attachment, Tukey’s honestly significant difference post hoc tests indicated that the HTR group had higher scores than other groups (*p* < 0.001) except for the HTFR (*p* = 0.594) or the average (*p* = 0.052) group‚ and the HTFR group scored higher than the average group (*p* < 0.001) (Fig. 4).

Regarding health problems due to excessive food reward pursuing, dogs in the LTFR group were rated lower than those in the High food responsive (HFR, *p* = 0.009) and HTFR (*p* = 0.004) groups. The HFR group was rated as having more eating-related health problems than the HTR (*p* = 0.002) group, while there was no difference between HFR and HTFR (*p* = 0.996) or average (*p* = 0.584) groups (Fig. 5). As for health problems due to excessive ball/toy reward pursuing HTR dogs were rated higher than HFR (*p* < 0.001) and average (*p* < 0.001) ones but did not differ from HTFR dogs (*p* = 0.962), while the latter were rated higher than average dogs (*p* < 0.001) (Fig. 5).

In terms of social problems due to excessive food reward pursuing, the HTR group was rated lower than both HFR and HTFR groups (all *p*s < 0.001). The HFR group did not differ from the HTFR (*p* = 0.993) but was higher than the average group (*p* < 0.001), and the HTFR group was rated also higher than the average group (*p* < 0.001) (Fig. 6). In terms of social problems due to excessive ball/toy reward pursuing, HTR dogs received higher scores than HFR (*p* < 0.001) and average dogs (*p* < 0.001) but did not differ from HTFR ones (*p* = 0.939). HTFR dogs were rated higher than average dogs (*p* = 0.001) (Fig. 6).

## Discussion

Results generally supported our hypotheses and indicated that there is individual variation in dogs’ responsiveness to food and ball/toy rewards as indexed by their owner-reported scores on the Canine Reward Responsiveness Scale (CRRS) and, in a subsample, by laboratory-observed behavioural correlates. This individual variation was related to the type of reward used during training, and high reward sensitivity was associated with relevant behavioural traits such as inattention, hyperactivity-impulsivity and indices of problems in physical health and social functioning.

Regarding *individual variation in dogs’ reward responsiveness*, a confirmatory factor analysis revealed that the items of our original question set represented two conceptually distinct phenomena, i.e., food and ball/toy responsiveness. Ten of the 15 Food responsiveness items were retained in a best-fitting model, compared to 14 of the 15 Ball/toy responsiveness items. This might either indicate that our question set defines the food responsiveness trait less lucidly than ball/toy responsiveness trait, as expression of the former may manifest itself in behaviours and circumstances that are additional to those described in the items. Alternatively, food responsiveness may be a less heterogeneous, multi-faceted trait than ball/toy responsiveness and thus describable with fewer items.

As noted, in research with humans, rewards are generally categorized along one of two broad categories, material (e.g., food, money) and social (e.g., positive social contact)^44,45^. Not only are these two types of rewards differentially implicated in relevant psychiatric disorders^46,47^, there are differences in ways in which atypically developing youth differ from their typically developing counterparts. For example, in neurotypical youth (i.e., without a neurodevelopmental disorder), monetary and social losses induce similar activity in the brain’s reward system. Whereas in children with ADHD, deficits in reward processing affect early brain signatures of both monetary and social losses, in children with ASD, deficits in reward processing affect early brain signatures of social losses only^48^. As such, although there may be similar etiological or neural mechanisms underlying material and social reward responsiveness^44^, these predispositions may function differently depending on the organization of the system in which they operate and yield behaviourally differentiated, and relatively heterogeneous phenotypes, i.e., multifinality^49^. In the current case of the CRRS item contents, both food and ball/toy rewards are at least partly material, while the ball/toy reward conceptualization inevitably incorporates social elements as well. Although it has been claimed that most companion dogs prefer using objects in interactions with people rather than playing asocially^50^, some dogs do engage in solitary object play; the motivation for play associated with a context that is primarily social may be quite different from that associated with solitary play^51^. Specifically, solitary object play appears to be both motivationally and structurally related to predatory behaviour^51^. As such, prior data with dogs is consistent with findings with humans suggesting that at some level, material and social rewards are differentially represented. Yet, it is not possible to determine the degree to which ball/toy rewards as conceptualised in our study represent material vs. social rewards, as we have no information about the extent to which ball/toy-directed approach and consummatory-like behaviours (e.g., chasing, manipulating, possessing the object itself) are rewarding or the extent to which behaviours that involve social interaction with the human partner (e.g., the human partner throws the ball/toy that enables the dog to chase it, the human partner may chase the dog to obtain the ball/toy, etc.) are rewarding.

In terms of descriptive characterization of adult dogs with regard to reward responsiveness, our results are consistent with findings indicating that individual differences in reward pursuing reflect distinct physiological and behavioural traits both in humans and in animals^52^ and that perception of the incentive effects of rewards varies considerably^53^. Breed differences that have already been identified in personality traits^54^ can be linked to Ball/toy responsiveness and playfulness (and chase proneness)^55,56^. The finding that retrievers were rated higher than all other breeds on Food responsiveness is consistent with prior results identifying this breed group as being at high-risk for obesity^57^ and also with reports from the human literature that suggest an association between reward sensitivity, food craving and relative body weight^58^.

Regarding *relationship to behaviour*, it is important to first note that the behavioural indices of our laboratory reward responsiveness paradigm have all been shown to be related to approach and consummatory behaviours inducible by rewards^59^. Although the current results generally evince convergent validity of the CRRS, our Ball/toy responsiveness (B/TR) measures exhibited stronger correlations with each other than our Food responsiveness (FR) measures. This might indicate that B/TR is relatively consistent across different contexts and settings, whereas FR may be more context-specific. On the other hand, dogs higher on Food responsiveness returned more frequently to the location of food consumption, which might be related to functions of reward relevant to learning^59^ thus reflecting increased expectations of finding additional rewards. Higher CRRS FR was also behaviourally reflected in decreased latency to approach and eat the food, possibly because more food-responsive dogs might have either better scent detecting capacities, or simply have more attentional bias to such stimuli. This explanatory hypothesis warrants further investigation. That dogs had more opportunity to gain reward-related interactive experience with the ball/toy than with the food during the first episode could have further influenced their reward-directed behaviours in the second episode (i.e., approach latency, time spent near the reward and return frequency correlated only with B/TR scores).

It is an important consideration that there was some overlap between aspects of the CRRS factors and those behavioural indices that correlated with each respective factor score, including time spent focusing on the unattainable reward and manipulating the apparatus to obtain the unattainable reward. These seem to reflect reward responsiveness independently of reward type. These findings are consistent with prior ones on dogs indicating that, in a two-choice food preference test, similar behaviours directed towards inaccessible food were associated with reward type preference^60^. Similarly, the findings of human studies suggest a link between trait approach motivation and attentional focus^61^, and between excessive reward consumption (i.e., substance-use disorders) and attentional bias for reward-related stimuli, with this bias directly proportional to magnitude of consumption^62^.

In relation to the observed association between *reward responsiveness and relevant behavioural traits*, our findings generally evince concurrent validity of the CRRS. We found an overall positive linear relationship between the first hyperactivity-impulsivity subscale (reflecting behaviours related to barking endlessly and difficulty with maintaining stay and waiting) and B/TR. In addition, at different levels of the first hyperactivity-impulsivity subscale, dogs who exhibited increases in motivation by/for reward (any type) during training scored higher on B/TR. On one hand this may indicate that training can influence dogs’ B/TR (see the above noted link between an increase in reward-motivation during training and higher B/TR) and that its effect on reward-motivation is a function of their activity/impulsivity. On the other hand, this may also suggest that dogs whose reward-motivation is less influenced by training (e.g. because of their activity/impulsivity) are also less ball/toy responsive. As with any other-report rating scale, a limitation nevertheless is that owners’ responses on the CRRS could have been biased by their idea of an ideal companion dog (as opposed to their actual dog) and they may have highlighted positive behaviours over negative ones. The degree to which owners are biased by these types of cognitions or heuristics is an important next step to examine in future research.

Interestingly, the frequency with which dogs received rewards (treats or ball/toy) for obedience tasks was related to both B/TR and FR, however, in opposite directions. An increase in rewarding frequency for obedience was paralleled by an increase in B/TR scores, but by a decrease in FR scores. Possibly this finding might be due to the confounding effects of owners’ time spent with their dogs, i.e., an increase in rewarding frequency likely corresponds to an increased amount of time actively spent with the dog. Increased engagement in joint activities might lead to higher levels of mental and physical satisfaction and thus less food pursuing behaviour, which, in some cases, might serve as a replacement activity in the face of boredom.

There was a negative association between dogs’ Inattention and B/TR. This association is comparable to human findings on the association between inattention in ADHD and dysfunctional reward processing, with that association hypothetically being related to or resulting from drowsiness, forgetfulness, lethargy, passivity, or sluggish cognitive tempo, key features of the Inattentive subtype of the disorder^63^. Others have argued that, a tendency to automatically attend to reward-related stimuli is key to appraising the incentive salience of cues or stimuli. It stands to reason that in youth with ADHD, inattention may disrupt the ability to process the incentive properties of reward-related stimuli, which could in turn result in deficits in reward responsiveness^63^.

A negative relationship between Inattention and B/TR was seen in all dogs and the strength of this association was influenced by Training method. The association was strongest in dogs who were rewarded by play (ball/toy) or social reinforcement and weakest in dogs rewarded only by food during training. Possibly, using only food reward during training goes together with generally less ball/toy usage, in turn attenuating the expression of a relationship between inattention and B/TR. We have to note, however, that dogs rewarded only by play or social reinforcement are underrepresented in our sample as compared the other groups, so this result has to be interpreted with caution. It would still be worth investigating in more detail how and why dogs’ attentional abilities and B/TR (and FR) are associated, since these findings could have interesting and useful implications for dog training.

Dogs’ Hyperactivity-impulsivity and Inattention scores were found to play key roles in predicting extreme versus average Ball/toy and Food responsiveness, and the relationship between reward responsiveness traits and Reward frequency and Training method were more relevant in these extreme groups than in the average group. Since direction of cause-and-effects remain unclear, different explanatory hypotheses are viable. A rare frequency of rewarding might contribute to increased hyperactivity-impulsivity and inattention traits in dogs with extreme *low* Ball/toy and Food responsiveness, and to increased hyperactivity-impulsivity traits in dogs with extreme *high* Ball/toy and *low* Food responsiveness. However, it might be the other way around; rare rewarding paired with greater hyperactivity-impulsivity and inattention might result in extreme high Ball/toy and low Food responsiveness. We also found that, although overall, Inattention was negatively associated with B/TR, dogs with greater Hyperactivity-impulsivity *paired with* greater Inattention were more likely to be highly responsive to both reward types. Interestingly, boys without ADHD exhibit a response bias toward a more frequently rewarded behavioural alternative, irrespective of which alternative they were last rewarded on, whereas boys with ADHD showed different patterns of response bias wherein they exhibited a bias toward the alternative they were last rewarded on. As such, relative to typically developing peers, who exhibit a response bias reflective of their reinforcement history, youth with ADHD are more sensitive to individual instances of reward^64^ suggesting some support for the latter conceptualization.

Taken together, these results are consistent with previous findings about the link between hyperactivity-impulsivity, inattention, and reward responsiveness in humans and animals. Although pathological levels of hyperactivity-impulsivity and inattention have not been unequivocally established in dogs, hyperactivity can be considered a behaviour problem that requires treatment by veterinary behaviourists^65^. Indeed, there are case studies describing the veterinary treatment of ADHD in dogs^66^. In humans, impulsivity has been shown to be associated with increased reward-seeking behaviour^67^, as well as with vulnerability to development of addictive diseases, which correspond to pathologically high levels of reward seeking^68,69^. Finally, the hyperactivity-impulsivity symptoms of ADHD are associated with dysfunctional reward processing^70^.

As one of our final aims, we examined the association between reward responsiveness and *problems in physical health and social functioning*. In humans, substance use disorders are defined as uncontrollable and compulsive substance seeking and usage that persists in spite of negative health and social consequences^2^, and some have argued that the development of addiction reflects a shift from impulsivity to compulsivity^17^. We found positive associations between extreme high reward responsiveness and increased likelihoods of having problems with physical health and/or social functioning (attributable to excessive pursuit of rewards). Additionally, extreme high responsiveness to both types of rewards was related to higher levels of toy/object attachment. Although pathological levels of food and/or ball/toy reward responsiveness have not yet been established in dogs, excessive preoccupation with an object or toy is among the criteria for Canine Compulsive Disorder (CCD), a behavioural syndrome that is purportedly a good candidate animal model of human obsessive-compulsive disorders^71^. Although it is obviously beyond the scope of the CRSS to serve as a diagnostic tool for behavioural abnormalities, these results suggest that extreme high levels of reward responsiveness could have negative consequences and, as such, that it is an issue that needs further attention in canine research.

Our results have implications for both the training and the welfare of domestic dogs. In general, natural rewards (such as food consumption, social play) contribute to increased welfare status as these are associated with a pleasurable inner state in animals and in humans^24,59,72^. Conversely, excessive pursuit of rewards implies impaired welfare status^25^. Since some of our findings indicate that there is a possibility of developing behavioural problems or disorders in part due to reward responsiveness, questions related to whether this characteristic confers a risk for abnormalities should be taken seriously and investigated in more detail. The CRRS may provide a good basis for further examination of either the genetic contributions (e.g. potential high-risk groups, such as retrievers for obesity, or hyperactive-impulsive individuals), or the influence of several aspects of the training method (e.g., reward-type used, rewarding frequency, etc.) on the variability of the represented reward pursuing behaviours in dogs. Given its relationship to both observed behaviour relevant to reward responsiveness and to characteristics that are relevant indirectly (activity and attention levels) and directly (increase in motivation and differences in relation to training method) to predicting response to training, the CRRS is a promising screening tool for dog trainers and behaviour specialist veterinarians. For example, trainers may be able to use it to better tailor training approaches to individual dogs (“personalized medicine”), given a dog’s scores on CRRS factors. In light of data indicating that, in humans, reward responsiveness is malleable^73^, it stands to reason that it is also changeable or malleable in dogs. Our results indicate that, at extreme levels, reward responsiveness is associated with increased likelihood of problems with physical and/or social problems and, as such, reward responsiveness should be examined in future studies as a potential mechanism of change in mitigating these problems in dogs.

## Conclusion

Taken together, the results of the present study are evidence of reliability and validity of a newly developed owner-report measure of canine reward responsiveness. Findings also suggest individual variability in reward responsiveness in domestic dogs and that such variability has implications for differences in relevant behavioural traits such as inattention, hyperactivity-impulsivity and with indices of welfare, i.e., problems with physical health and social functioning. Not only is this pattern of results encouraging with regard to usefulness of the CRRS in measuring reward responsiveness in dogs‚ but also provides early evidence of the domestic dog as a reliable and valid animal model of human behaviour; in the present case, of processing material and social rewards.

## Methods

### Questionnaire development

Our aim in developing the Canine Reward Responsiveness Scale (CRRS) was to create a measure along which dogs can be characterized given the intensity of their everyday ball/toy and food pursuing behavioural characteristics. Item generation was informed by our experience with dog behaviour, available questionnaires measuring such behaviour^27,28^, professional dog trainers’ experience with dogs’ reward-related behaviour in different contexts, as well as the empirical literature (both human and animal). Regarding the empirical literature, we primarily relied on investigations focused on neurobiological, physiological and psychological correlates of behaviour in response to natural rewards and drugs of abuse^23,25^, reward processing, including reward cue approach motivation^1,61,74^, the development and maintenance of psychiatric disorders relevant to excessive pursuit of rewards^25,75^ or excessive involvement in particular activities^16,76–78^, rating scale measures of psychoactive substance and behavioural/nonsubstance addictions in humans^79,80^ and, finally, on studies of the development and validation of rating scale measures of specific behavioural traits in dogs^27,29,37^.

The final pool of items are 15–15 statements referring to behavioural manifestations of the dog’s ball/toy and food responsiveness tendencies, respectively. Items reflect the: i) amount and frequency of reward consumption and reward seeking behaviour; ii) motivation to obtain rewards; iii) level of excitement prior to and during reward consumption; iv) reaction to withdrawal of the expected reward; v) extent of focused engagement in the rewarding activity; vi) reward preferences. The statements, which also comprise descriptors of affect or mood, describe the dog’s responses to rewarding stimuli in specific situations. These are formulated in a way to be readily applicable to everyday situations in a general domestic setting. The items are to be rated along a 5-point Likert scale, reflecting the extent to which the owner disagrees or agrees with the statement (1 – *strongly disagree*; 2 – *partly disagree*; 3 – *neither agree nor disagree*; 4 – *partly agree*; 5 – *strongly agree*). Data on the owner’s opinion about the dog’s behaviours reflective of reward pursuing in general, attachment to any favourite object (e.g. ball, toy), and health- and social (i.e., owner-dog conflict) problems as well as on basic demographic characteristics and dog training history were also collected. For the complete CRRS and the additional questions, see Supplementary Text S1.

After finalization – based on a first set of answers and feedbacks as part of preliminary data collection – the questionnaire was distributed over the internet in three languages (English, German, and Hungarian). To ensure that there are no differences in responses that are a result of inadequate translation, the original, Hungarian questionnaire was translated into English and German and then back-translated (both by native speakers), following best practice guidelines for questionnaire translation and back-translation^81^.

### Participants

In total, 2149 responses were obtained (in English: *n* = 610, in German: *n* = 704, from Germany; in Hungarian: *n* = 835, from Hungary). The majority of respondents were female (*n* = 1923) owners (*M*_age_: 39.65 years, *SD* = 12.39), who, in case of English-speaking participants, were from 19 different countries (mostly USA *n* = 134 and the UK *n* = 39, with unknown *n* = 380).

The rated dogs represented 197 different breeds and the majority were purebred (*n* = 1417) but a considerable portion were mixed (*n* = 379) and cross-breed (*n* = 353). Categorization of purebred dogs into breed groups was based on genetic relatedness^82^ and were as follows: basal breeds (*n* = 42), guard dogs (*n* = 117), herding dogs (*n* = 334), mastiffs (*n* = 119), retrievers (*n* = 179), scent dogs (*n* = 200), sighthounds (*n* = 63), small terriers (*n* = 91), toy breeds (*n* = 131) and ungrouped purebreds (*n* = 141). Dogs’ mean age was 5.41 years (*SD* = 3.22) and the sample represented each of four sex categories (males: *n* = 542, females: *n* = 488, neutered males: *n* = 527, neutered females: *n* = 594). Most were kept as companion animals (*n* = 1448), some for hobby-related reasons, e.g. sports (*n* = 512) and some as professional working dogs (*n* = 189). Training status was defined as: not trained (*n* = 27), trained by the owner (*n* = 722), trained in dog school (*n* = 938), trained privately by professional trainer (*n* = 218), and has special training certificate (*n* = 244). During training, the following types of rewards were used: food only (*n* = 735), food as part of clicker training (*n* = 459), ball/toy only (*n* = 68), food *and* ball/toy (*n* = 789), and social reinforcement only (*n* = 63).

### Questionnaire validation

Since the set of items was specifically developed to contain two subscales (i.e., Ball/toy and Food responsiveness), evidence of internal validity was evaluated using Confirmatory Factor Analysis. The resultant factors were assessed for evidence of internal consistency (considered acceptable if α > 0.70)^83^. Evidence of convergent validity was evaluated via bivariate correlations between the resultant factor scores and independent items reflecting the owner’s opinion about the dog’s behaviours reflective of reward pursuing in general (see Text S1).

For purposes of external validation, owners were also asked to fill out a psychometrically validated rating scale of dog attention and activity/impulsivity^37^ to assess evidence of concurrent validity. The subscales of this measure are comprised of items describing i) in case of the attention subscale (IA) – to what extent a dog exhibits difficulties with concentration and performing practiced tasks, is easily distractible and loses interest, and does not pay attention to someone directly speaking to him/her; ii) in case of the first activity/impulsivity subscale (HY1) – to what extent a dog barks endlessly, is in constant motion and/or fidgets, has difficulty maintaining stay and with waiting; iii) in case of the second activity/impulsivity subscale (HY2) – to what extent a dog enjoys active play/running around, reacts hastily, and is in constant motion^37^.

In addition, behavioural data in a controlled laboratory setting was collected from two subsamples of dogs in brief paradigms each, designed to measure canine reaction to presentation of ball/toy (*n* = 30, 17 males, *M*_age_ = 3.98; *SD* = 2.81, from 16 different breeds and mixed breeds) and food (*n* = 30, 15 males, *M*_age_ = 4.13; *SD* = 2.42, from 14 different breeds and mixed breeds) rewards. The tests took place in a 6.4 m × 5.2 m laboratory room at the Department of Ethology at Eötvös Loránd University, Budapest, Hungary, between July 2016 and June 2017. They consisted of two consecutive 2-minute episodes, during which the dog, the owner and a female experimenter were present. Dogs were allowed to behave freely without any external control and thus the owner and experimenter remained passive during both episodes. In the first episode, the dog was presented with a freely available and attainable reward (i.e., either a piece of sausage or the dogs’ favourite ball/toy) placed in the middle of the room in the opening of a cage. During the second episode, which followed the first one after a ~30-second interruption, the reward – presented at the same location – was unattainable (i.e. inside the closed cage). Behaviour was video recorded and data were subsequently analysed with Solomon coder (© András Péter, http://solomoncoder.com). For detailed description of the experiment and definitions of variables used to examine evidence of convergent validity – i.e., bivariate correlations – between the CRRS and observable behaviour in a laboratory reward responsiveness paradigm see Table 2, Supplementary Text S3 and Supplementary Table S4.

### Ethical statement

Owners volunteered to participate in the behavioural paradigms with their dogs upon filling out the CRRS, and gave written consent. Non-invasive animal research is currently allowed without need for permission from the University Institutional Animal Care and Use Committee (UIACUC, Eötvös Loránd University, Hungary). A written statement (#PEI/001/3819-4/2015) was obtained from the Food Chain Safety and Animal Health Directorate Government Office based on the decision of the Scientific Ethic Council of Animal Experiments. According to this statement and the corresponding definition by law, the current non-invasive observational study is not an animal experiment.

### Analytic Plan

SPSS V22.0.0.0 was used for all analyses. Confirmatory factor analysis was conducted in AMOS. Both modification indices and the items’ estimated standardized factor loadings were referenced. Model fit was examined using the *X*^2^/*df* ratio, root mean square error of approximation (RMSEA), and the comparative fit index (CFI). Conventionally, a *X*^2^/*df* ratio of 5:1^84,85^, a RMSEA ≤0.10, and a CFI >0.90 indicate sufficient fit^86^ and *X*^2^/*df* ratio of 2, RMSEA ≤0.06, and CFI >0.95 indicate excellent fit^87^.

Factor scores for each dog were calculated in accordance with the distribution of the final items across the two factors, by taking the mean item rating of all items belonging to the respective factor.

The main and interaction (all two-way of non-demographic variables) effects of independent variables (IVs: IA, HY1, HY2 [calculated following]^37^; Breed; Training method; Toy/object attachment; Reward frequency; and Increase in motivation – see Supplementary Table S5) on dependent variables (DVs: Food and Ball/toy responsiveness) of interest were examined in a multivariate general linear model (GLM). All non-significant interactions were removed from the model, using backward elimination, and the model was re-run until there were no non-significant terms. To characterize our sample using the CRRS, with regard to reward responsiveness, we created four extreme groups (i.e., with scores ≥ 1 *SD* ± the sample mean) based on Ball/toy and Food responsiveness scores. The groups were Group 1, low on Ball/toy and Food responsiveness (LTFR), Group 2, high on Ball/toy and low on Food responsiveness (HTR), Group 3, low on Ball/toy and high on Food responsiveness (HFR), and Group 4, high on both Ball/toy and Food responsiveness (HTFR). From among dogs with CRRS scores within ± 1 *SD* the sample mean on both factors (*n* = 921), a random group of dogs (*n* = 100) was selected to create the reference group, Group 5 (ATFR). A multinomial logistic regression analysis was performed to predict likelihood of membership in any of the four extreme groups, given the following IVs: IA, HY1, HY2, Training method, Reward frequency, and Increase in motivation and a MANOVA to compare groups on Toy/object attachment and problems with i) physical health due to excessive a) eating and b) playing with ball/toys and ii) social functioning (i.e., owner-dog conflict due to c) eating and d) ball/toy playing habits) – for variable definitions see Supplementary Table S5.

The datasets generated and/or analysed during the current study are available from the corresponding author upon reasonable request.

## Electronic supplementary material

Supplementary Information

## References

[1] National Institute of Mental Health. *Positive Valence Systems: Workshop Proceedings* (2011).

[2] Baskin-Sommers, A. R. & Foti, D. Abnormal reward functioning across substance use disorders and major depressive disorder: Considering reward as a transdiagnostic mechanism. *International Journal of Psychophysiology*10.1016/j.ijpsycho.2015.01.011 (2014). 10.1016/j.ijpsycho.2015.01.011 25655926

[3] Tripp G, Wickens JR (2008). Research review: Dopamine transfer deficit: A neurobiological theory of altered reinforcement mechanisms in ADHD. Journal of Child Psychology and Psychiatry and Allied Disciplines.

[4] Scott-Van Zeeland AA, Dapretto M, Ghahremani DG, Poldrack RA, Bookheimer SY (2010). Reward processing in autism. Autism Res..

[5] White SF (2013). Disrupted expected value and prediction error signaling in youths with disruptive behavior disorders during a passive avoidance task. Am. J. Psychiatry.

[6] Matthys W, Vanderschuren LJMJ, Schutter DJLG (2012). The neurobiology of oppositional defiant disorder and conduct disorder: Altered functioning in three mental domains. Dev. Psychopathol..

[7] Luman M, Van Meel CS, Oosterlaan J, Sergeant JA, Geurts HM (2009). Does reward frequency or magnitude drive reinforcement-learning in attention-deficit/hyperactivity disorder?. Psychiatry Res..

[8] Baxter AJ (2015). The epidemiology and global burden of autism spectrum disorders. Psychol. Med..

[9] Maughan ,B, Rowe R, Messer J, Goodman R, Meltzer H (2004). Conduct disorder and oppositional defiant disorder in a national sample: developmental epidemiology. J. Clin. Child Psychol. Psychiatry.

[10] Spencer TJ, Biederman J, Mick E (2007). Attention-deficit/hyperactivity disorder: diagnosis, lifespan, comorbidities, and neurobiology. J. Pediatr. Psychol..

[11] Bunford, N., Evans, S. W. & Langberg, J. M. Emotion Dysregulation Is Associated With Social Impairment Among Young Adolescents With ADHD. *J. Atten. Disord*. 10.1177/1087054714527793 (2014). 10.1177/1087054714527793 24681899

[12] Bunford N, Evans SW, Wymbs F (2015). ADHD and Emotion Dysregulation Among Children and Adolescents. Clinical Child and Family Psychology Review.

[13] Le HH (2014). Economic impact of childhood/adolescent ADHD in a European setting: the Netherlands as a reference case. Eur. Child Adolesc. Psychiatry.

[14] van der Staay FJ, Arndt SS, Nordquist RE (2009). Evaluation of animal models of neurobehavioral disorders. Behav. brain Funct..

[15] Bunford N, Andics A, Kis A, Miklósi Á, Gácsi M (2017). Canis familiaris as model for non-invasive comparative neuroscience. Trends Neurosci..

[16] Potenza MN (2009). The Importance of Animal Models of DecisionMaking, Gambling and Related Behaviors: Implications for Translational Research in Addiction. Neuropsychopharmacology.

[17] Belin D, Mar AC, Dalley JW, Robbins TW, Everitt BJ (2008). High Impulsivity Predicts the Switch to Compulsive Cocaine-Taking. Science (80-.)..

[18] Zeeb FD, Robbins TW, Winstanley CA (2009). Serotonergic and Dopaminergic Modulation of Gambling Behavior as Assessed Using a Novel Rat Gambling Task. Neuropsychopharmacology.

[19] Aston-Jones G, Smith RJ, Moorman DE, Richardson KA (2009). Role of lateral hypothalamic orexin neurons in reward processing and addiction. Neuropharmacology.

[20] Berridge KC, Robinson TE, Aldridge JW (2009). Dissecting components of reward:‘liking’,‘wanting’, and learning. Curr. Opin. Pharmacol..

[21] Flagel SB, Akil H, Robinson TE (2009). Individual differences in the attribution of incentive salience to reward-related cues: Implications for addiction. Neuropharmacology.

[22] Johnson PM, Kenny PJ (2010). Dopamine D2 receptors in addiction-like reward dysfunction and compulsive eating in obese rats. Nat. Neurosci..

[23] Alcaro A, Panskepp J (2011). The SEEKING mind: primal neuro-affective substrates for appetitive incentive states and their pathological dynamics in addictions and depression. Neurosci. Biobehav. Rev..

[24] Berridge KC, Kringelbach ML (2008). Affective neuroscience of pleasure: Reward in humans and animals. Psychopharmacology.

[25] Parylak SL, Koob GF, Zorrilla EP (2011). The dark side of food addiction. Physiol. Behav..

[26] Miklósi Á, Topál J (2013). What does it take to become ‘best friends’? Evolutionary changes in canine social competence. Trends Cogn. Sci..

[27] Vas J, Topál J, Pech E, Miklósi Á (2007). Measuring attention deficit and activity in dogs: A new application and validation of a human ADHD questionnaire. Appl. Anim. Behav. Sci..

[28] Kubinyi E, Pongrácz P, Miklósi Á (2009). Dog as a model for studying conspecific and heterospecific social learning. Journal of Veterinary Behavior: Clinical Applications and Research.

[29] Wright HF, Mills DS, Pollux PMJ (2011). Development and Validation of a Psychometric Tool for Assessing Impulsivity in the Domestic Dog (Canis familiaris). Int. J. Comp. Psychol..

[30] Miklósi, Á. *Dog Behaviour Evolution and Cognition*. (Oxford University Press, 2014).

[31] Head E (2013). A canine model of human aging and Alzheimer’s disease. BBA - Mol. Basis Dis..

[32] Range F, Horn L, Viranyi Z, Huber L (2009). The absence of reward induces inequity aversion in dogs. Proc. Natl. Acad. Sci. USA.

[33] Wright HF, Mills DS, Pollux PMJ (2012). Behavioural and physiological correlates of impulsivity in the domestic dog (Canis familiaris). Physiol. Behav..

[34] Müller, C. A., Riemer, S., Virányi, Z., Huber, L. & Range, F. Inhibitory control, but not prolonged object-related experience appears to affect physical problem-solving performance of pet dogs. *PLoS One* **11**, (2016). 10.1371/journal.pone.0147753 PMC4749342 26863141

[35] Tauzin T, Kovács K, Topál J (2016). Dogs identify agents in third-party interactions on the basis of the observed degree of contingency. Psychol. Sci..

[36] Fugazza C, Miklósi Á (2015). Social learning in dog training: the effectiveness of the Do as I do method compared to shaping/clicker training. Appl. Anim. Behav. Sci..

[37] Lit, L., Schweitzer, J. B., Iosif, A. M. & Obermauer, A. M. Owner reports of attention, activity and impulsivity in dogs: a replication study. *Behav. Brain Funct*. **6**, (2010). 10.1186/1744-9081-6-1 PMC2823640 20047681

[38] Evans SW, Owens JS, Bunford N (2014). Evidence-Based Psychosocial Treatments for Children and Adolescents Evidence-Based Psychosocial Treatments for Children and Adolescents With Disruptive Behavior. J. Clin. child Adolesc. Psychol..

[39] Kazdin, A. E. Problem-solving skills training and parent management training for oppositiona defiant disorder and conduct disorder. *Evidence-based psychotherapies for children and adolescents (2nd ed.)*. 211–226 (2010).

[40] Rooney NJ, Cowan S (2011). Training methods and owner–dog interactions: Links with dog behaviour and learning ability. Appl. Anim. Behav. Sci..

[41] Hiby EF, Rooney NJ, Bradshaw JWS (2004). Dog training methods: Their use, effectiveness and interaction with behaviour and welfare. Anim. Welf..

[42] Blackwell EJ, Twells C, Seawright A, Casey RA (2008). The relationship between training methods and the occurrence of behavior problems, as reported by owners, in a population of domestic dogs. J. Vet. Behav. Clin. Appl. Res..

[43] Sheppard G, Mills DS (2002). The development of a psychometric scale for the evaluation of the emotional predispositions of pet dogs. Int. J. Comp. Psychol..

[44] Izuma K, Saito DN, Sadato N (2008). Processing of Social and Monetary Rewards in the Human Striatum. Neuron.

[45] Lin A, Adolphs R, Rangel A (2012). Social and monetary reward learning engage overlapping neural substrates. Soc. Cogn. Affect. Neurosci..

[46] Lin, A., Rangel, A. & Adolphs, R. Impaired learning of social compared to monetary rewards in autism. *Front. Neurosci*. 10.3389/fnins.2012.00143 (2012). 10.3389/fnins.2012.00143 PMC3461406 23060743

[47] Kohls G, Herpertz-Dahlmann B, Konrad K (2009). Hyperresponsiveness to social rewards in children and adolescents with attention-deficit/hyperactivity disorder (ADHD). Behav Brain Funct..

[48] Gonzalez-Gadea ML (2016). Neural markers of social and monetary rewards in children with Attention-Deficit/Hyperactivity Disorder and Autism Spectrum Disorder. Sci. Rep..

[49] Cicchetti D, Rogosch FA (2002). A developmental psychopathology perspective on adolescence. J. Consult. Clin. Psychol..

[50] Pullen AJ, Merrill RJN, Bradshaw JWS (2010). Preferences for toy types and presentations in kennel housed dogs. Appl. Anim. Behav. Sci..

[51] Bradshaw JWS, Pullen AJ, Rooney NJ (2015). Why do adult dogs ‘play’?. Behav. Processes.

[52] Dalley JW (2007). Nucleus accumbens D2/3 receptors predict trait impulsivity and cocaine reinforcement. Science (80-.)..

[53] Mason WA, Sharpe LG, Saxon SV (1963). Preferential responses of young chimpanzees to food and social rewards. Psychol. Rec..

[54] Turcsán B, Kubinyi E, Miklósi Á (2011). Trainability and boldness traits differ between dog breed clusters based on conventional breed categories and genetic relatedness. Appl. Anim. Behav. Sci..

[55] Svartberg K, Forkman B (2002). Personality traits in the domestic dog (Canis familiaris). Appl. Anim. Behav. Sci..

[56] Svartberg K (2005). A comparison of behaviour in test and in everyday life: Evidence of three consistent boldness-related personality traits in dogs. Appl. Anim. Behav. Sci..

[57] Lund EMEEME, Armstrong PJ, Kirk Ca, Klausner JS (2006). Prevalence and Risk Factors for Obesity in Adult Dogs from Private US Veterinary Practices. J. Appl. Res. Vet. Med..

[58] Franken IHA, Muris P (2005). Individual differences in reward sensitivity are related to food craving and relative body weight in healthy women. Appetite.

[59] Schultz W (2004). Neural coding of basic reward terms of animal learning theory, game theory, microeconomics and behavioural ecology. Current Opinion in Neurobiology.

[60] Thompson H, Riemer S, Ellis SLH, Burman OHP (2016). Behaviour directed towards inaccessible food predicts consumption-A novel way of assessing food preference. Appl. Anim. Behav. Sci..

[61] Gable PA, Harmon-Jones E (2008). Approach-motivated positive affect reduces breadth of attention: Research article. Psychol. Sci..

[62] Field M, Cox WM (2008). Attentional bias in addictive behaviors: A review of its development, causes, and consequences. Drug and Alcohol Dependence.

[63] Meinzer MC, Pettit JW, Leventhal AM, Hill RM (2012). Explaining the Covariance Between Attention-Deficit Hyperactivity Disorder Symptoms and Depressive Symptoms: The Role of Hedonic Responsivity. J. Clin. Psychol..

[64] Tripp G, Alsop B (1999). Sensitivity to Reward Frequency in Boys with Attention Deficit Hyperactivity Disorder. J. Clin. Child Adolesc. Psychol..

[65] *Behavior Problems of the Dog and Cat*. (Elsevier Health Sciences, 2012).

[66] Piturru P (2014). Methylphenidate use in dogs with attention deficit hyperactivity disorder (ADHD). *Tierärztliche Prax*. Kleintiere.

[67] Monterosso J, Ainslie G (1999). Beyond discounting: Possible experimental models of impulse control. Psychopharmacology.

[68] Kreek MJ, Nielsen DA, Butelman ER, LaForge KS (2005). Genetic influences on impulsivity, risk taking, stress responsivity and vulnerability to drug abuse and addiction. Nat. Neurosci..

[69] Dom G, D’Haene P, Hulstijn W, Sabbe B (2006). Impulsivity in abstinent early- and late-onset alcoholics: Differences in self-report measures and a discounting task. Addiction.

[70] Strohle A (2008). Reward anticipation and outcomes in adult males with attention-deficit/hyperactivity disorder. Neuroimage.

[71] Dodman NH (2016). Genomic risk for severe canine compulsive disorder, a dog model of human OCD. Int. J. Appl. Res. Vet. Med..

[72] Panksepp J (2005). Affective consciousness: Core emotional feelings in animals and humans. Conscious. Cogn..

[73] Prause, N., Siegle, G. J., Deblieck, C., Wu, A. & Iacoboni, M. EEG to primary rewards: Predictive utility and malleability by brain stimulation. *PLoS One* **11** (2016). 10.1371/journal.pone.0165646 PMC5130195 27902711

[74] McNaughton N, Corr PJ (2004). A two-dimensional neuropsychology of defense: fear/anxiety and defensive distance. Neurosci. Biobehav. Rev..

[75] Hyman SE (2005). Addiction: A disease of learning and memory. American Journal of Psychiatry.

[76] Griffiths MA (2005). ‘components’ model of addiction within a biopsychosocial framework. J. Subst. Use.

[77] Holden C (2010). Behavioral Addictions Debut in Proposed DSM-V. Science (80-.)..

[78] Grant JE, Potenza MN, Weinstein A, Gorelick DA (2010). Introduction to behavioral addictions. Am J Drug Alcohol Abus..

[79] Conigrave KM, Hall WD, Saunders JB (1995). The AUDIT questionnaire: choosing a cut‐off score. Addiction.

[80] Kellogg SH (2003). The Kreek-McHugh-Schluger-Kellogg scale: A new, rapid method for quantifying substance abuse and its possible applications. Drug Alcohol Depend..

[81] Wild D (2005). Principles of good practice for the translation and cultural adaptation process for patient-reported outcomes (PRO) measures: report of the ISPOR task force for translation and cultural adaptation. Value Heal..

[82] von Holdt BM (2010). Genome-wide SNP and haplotype analyses reveal a rich history underlying dog domestication. Nature.

[83] Nunnally, J. C. *Psychometric Theory*. (McGraw-Hill, 1978).

[84] Hooper D, Coughlan J, Mullen MR (2008). Structural equation modelling: Guidelines for determining model fit. Electron. J. Bus. Res. Methods.

[85] Wheaton B, Muthen B, Alwin DF, Summers GF (1977). Assessing reliability and stability in panel models. Sociol. Methodol..

[86] Bentler PM (1990). Comparative fit indexes in structural models. Psychol. Bull..

[87] Hu L, Bentler PM (1999). Cutoff criteria for fit indexes in covariance structure analysis: Conventional criteria versus new alternatives. Struct. Equ. Model. A Multidiscip. J..

