# Supplementary material for: Development and validation of the Canine Reward Responsiveness Scale –Examining individual differences in reward responsiveness of the domestic dog
Source: Sci Rep. 2018 Mar 13;8:4421. doi: 10.1038/s41598-018-22605-1 (PMC5849691; doi:10.1038/s41598-018-22605-1)
Supplement: Supplementary file 1 — Supplementary Information [file 41598_2018_22605_MOESM1_ESM.docx]

**Supplementary Information for**

**Development and validation of the Canine Reward Responsiveness Scale –**

Examining individual differences in reward responsiveness of the domestic dog

**Author list and affiliations**

Linda Gerencsér^1*^, Nóra Bunford^1,2^, Alexandra Moesta^3^ and Ádám Miklósi^1,4^

^1^ Eötvös Loránd University, Institute of Biology, Department of Ethology, 1117 Budapest, Pázmány Péter sétány 1/C

^2^ Hungarian Academy of Sciences, Research Centre for the Natural Sciences, Institute of Cognitive Neuroscience and Psychology, 1117 Budapest, Magyar tudósok körútja 2

^3^ WALTHAM Centre for Pet Nutrition, Freeby Lane, Waltham-on-the-Wolds, Melton Mowbray, UK

^4^ MTA-ELTE Comparative Ethology Research Group, Budapest, Hungary

*Correspondence: [linda.gerencser@gmail.com](mailto:linda.gerencser@gmail.com) (L. Gerencsér)

**Text S1 The items of the dog reward responsiveness questionnaire**

***Items related to CRRS***

To be answered along a 1-5 Likert scale according to the following:

1. not characteristic at all
2. moderately uncharacteristic
3. partly characteristic, partly not
4. moderately characteristic
5. totally characteristic

My dog …

1. … behaves in a pushy/annoying way if he/she wants to get some food (e.g. stares, barks, jumps, paws, pokes with me his/her nose). (f1)^[[1]](#footnote-1)^*
2. … goes to unfamiliar people to beg for food. (f2)^*^
3. … wolfs down his/her food (e.g. eats up his/her portion strikingly fast, swallows the bites without chewing). (f3)^*^
4. … is responsive if he/she does not receive food at the usual time (e.g. starts to behave excitedly and /or indicates that he/she would like to eat). (f4) ^*^
5. … tends to initiate on his/her own if he/she wants to eat (e.g. asks for food even when I don’t have any food with me / in my hands). (f5)
6. … is so focused on eating the treat that he/she hardly notices what is happening around him/her. (f6) ^*^
7. … gets excited right away if he/she can get some food (e.g. gets frisky, pays a lot of attention when he/she expects to be fed). (f7) ^*^
8. … leaves leftover food in his/her bowl after the usual everyday feeding. (R) (f8) ^*^

To be answered along a 1-5 Likert scale according to the following:

1. disagree completely
2. disagree moderately
3. partly agree, partly disagree
4. agree moderately
5. agree completely

My dog …

1. … can be motivated by a treat or food to do things that he/she is not keen on doing or tolerating otherwise (e.g. approaching a person/object that is frightening to him/her, getting in the car, etc.). (f9)
2. … has a voracious appetite (e.g. would eat a lot more than he/she requires). (f10) ^*^
3. … can be easily distracted from eating (e.g. by sounds, activities, other dogs/people, etc.). (R) (f11)
4. … does not tend to indicate that he/she would like to eat. (R) (f12)
5. … shows interest in the food only when he/she is really hungry. (R) (f13) ^*^
6. … readily eats anything. (f14) ^*^
7. … has a favourite food. (f15)

To be answered along a 1-5 Likert scale according to the following:

1. not characteristic at all
2. moderately uncharacteristic
3. partly characteristic, partly not
4. moderately characteristic
5. totally characteristic

My dog …

1. … behaves in a pushy/annoying way if he/she wants to play ball or play with any other object (e.g. stares, barks, jumps, paws, pokes me with his/her nose indicating that I should throw the ball, stick, etc.). (b/t1)^[[2]](#footnote-2)^*
2. … initiates play with balls or with other objects even with people unfamiliar to him/her (e.g. puts the ball, stick, etc. in front of them indicating that they should throw it). (b/t2) ^*^
3. … runs very eagerly after the thrown ball / object. (b/t3) ^*^
4. … is responsive if he/she cannot play ball / play with other objects at the usual place and/or time (e.g. starts to behave excitedly, frustrated and /or indicates that he/she would like to play ball). (b/t4) ^*^
5. … takes the initiative on his/her own if he/she wants to play ball or play with other objects (e.g. brings the ball / toy to me even when I am doing something else). (b/t5) ^*^
6. … is so focused on playing ball / fetching objects that he/she hardly notices what is happening around him/her. (b/t6) ^*^
7. … gets excited right away if he/she can play ball or fetch other objects (e.g. gets frisky, pays a lot of attention, runs ahead, when he/she expects the ball to be thrown). (b/t7) ^*^
8. … quits playing ball / fetching other objects before I would stop the play. (R) (b/t8) ^*^

To be answered along a 1-5 Likert scale according to the following:

1. disagree completely
2. disagree moderately
3. partly agree, partly disagree
4. agree moderately
5. agree completely

My dog …

1. … can be motivated by a ball / toy to do things that he/she is not keen on doing or tolerating otherwise (e.g. approaching a person/object that is frightening to him/her, getting in the car, etc.). (b/t9) ^*^
2. … is tireless when it comes to playing ball / fetching objects. (b/t10) ^*^
3. … can be easily distracted from playing ball / fetching objects (e.g. by sounds, activities, other dogs/people, etc.). (R) (b/t11) ^*^
4. … does not tend to indicate that he/she would like to play ball or play with other toys. (R) (b/t12) ^*^
5. … only plays ball or fetches other objects when he/she is in a playful mood. (R) (b/t13) ^*^
6. … readily plays with any object. (b/t14) ^*^
7. … has a favourite toy or object. (b/t15)

***Other questions***

1. Owner’s age
2. Owner’s gender
3. Dog’s age
4. Dog’s breed
5. Dog’s gender
6. From the following options, my dog’s training level is best characterized as:^[[3]](#footnote-3)^**
   1. has not received any training;
   2. was/is mostly trained by myself;
   3. visited/visits training courses in a dog school;
   4. visited/visits dog school regularly;
   5. is/was trained individually by a professional dog trainer;
   6. has a special certificate, e.g. is a working-dog
7. What kind of reward method has been used predominantly during your dog’s training?^**^
   1. my dog has not been trained;
   2. treats;
   3. clicker training (clicker + treats);
   4. playing with a ball and/or other toys;
   5. social play without objects
8. How often do you practice obedience tasks with your dog and reward him/her with ball, toy or treat? (Any kind of task either at home, during a walk or as part of a direct training program.)
   1. never;
   2. a few times each month;
   3. at least once every week;
   4. a few times every week;
   5. almost every day;
   6. once every day;
   7. several times every day
9. It became more and more easy to motivate my dog with reward (e.g. treat, ball) during the course of the training.

1 - disagree completely

2 - disagree moderately

3 - partly agree, partly disagree

4 - agree moderately

5 - agree completely

1. To what extent is your dog attached to his/her favourite object?
   1. I do not know he/she does not have a favourite object;
   2. is not really attached to it; likes to play with it, likes it to be near him/her but does not look for it when it’s not there;
   3. notices if it’s not there, but looks for it only for a short time;
   4. is restless if it’s not there, looks for it till he/she finds it;
   5. is badly attached to it, it can hardly be taken away from him/her

To be answered along a 1-5 Likert scale according to the following:

1. not characteristic at all
2. moderately uncharacteristic
3. partly characteristic, partly not
4. moderately characteristic
5. totally characteristic
6. My dog had/has physical health-related problems as a consequence of his/her excessive eating (e.g. vomiting, diarrhoea, obesity, etc.).
7. My dog had physical health-related problems as a consequence of his/her excessive ball playing (e.g. accident, injury, etc.).

To be answered along a 1-5 Likert scale according to the following:

1. disagree completely
2. disagree moderately
3. partly agree, partly disagree
4. agree moderately
5. agree completely
6. My dog is obsessed with eating.
7. It annoys me that my dog likes to eat more than it should.
8. Sometimes I am angry with my dog because of his/her eating ’habits’.
9. My dog is obsessed with playing ball / fetching objects.
10. It annoys me that my dog likes to play ball / fetch objects frequently.
11. Sometimes I am angry with my dog because of his/her ball playing / object fetching ’habits’.

As part of the CRRS validation procedure (evidence of convergent validity), we used questions #13 and #16 above as independent items to check for correlations (Pearson’s *r*) with the Food responsiveness (FR) and Ball/toy responsiveness (B/TR) factors, respectively.

**Table S2 Estimates of Ball/toy and Food responsiveness at different levels of Breed and Toy/ball attachment**

| **Independent variable** | | **Ball/toy responsiveness (B/TR)** | | | | **Food responsiveness (FR)** | | | |
| --- | --- | --- | --- | --- | --- | --- | --- | --- | --- |
|  |  | Mean^a^ | Std. Error | 95% Confidence Interval | | Mean^a^ | Std. Error | 95% Confidence Interval | |
|  |  |  |  | Lower Bound | Upper Bound |  |  | Lower Bound | Upper Bound |
| **Breed** | Toy | 3.029 | .068 | 2.895 | 3.164 | 2.893 | .082 | 2.733 | 3.054 |
|  | sTerr | 3.189 | .081 | 3.030 | 3.348 | 2.788 | .097 | 2.598 | 2.978 |
|  | Basal | 2.646 | .117 | 2.416 | 2.875 | 2.692 | .140 | 2.418 | 2.965 |
|  | Herd | 3.321 | .048 | 3.226 | 3.415 | 2.844 | .058 | 2.731 | 2.957 |
|  | Mast | 3.099 | .071 | 2.961 | 3.237 | 2.976 | .084 | 2.811 | 3.141 |
|  | Retr | 3.307 | .059 | 3.191 | 3.424 | 3.383 | .071 | 3.244 | 3.522 |
|  | Sight | 3.061 | .099 | 2.867 | 3.254 | 2.888 | .118 | 2.657 | 3.118 |
|  | Scent | 3.147 | .056 | 3.036 | 3.258 | 3.065 | .067 | 2.933 | 3.197 |
|  | Guard | 3.224 | .070 | 3.087 | 3.361 | 2.866 | .083 | 2.702 | 3.029 |
|  | Purebr | 3.014 | .066 | 2.885 | 3.143 | 2.974 | .079 | 2.820 | 3.129 |
|  | Cross | 3.077 | .045 | 2.989 | 3.165 | 2.816 | .054 | 2.711 | 2.922 |
|  | Mixed | 3.163 | .046 | 3.073 | 3.254 | 2.867 | .055 | 2.759 | 2.976 |
| **Toy/object attachment** | 1 | 2.594 | .037 | 2.521 | 2.668 | 3.022 | .045 | 2.935 | 3.110 |
|  | 2 | 3.055 | .033 | 2.990 | 3.120 | 2.886 | .039 | 2.809 | 2.964 |
|  | 3 | 3.248 | .046 | 3.158 | 3.339 | 2.908 | .055 | 2.800 | 3.017 |
|  | 4 | 3.528 | .067 | 3.397 | 3.660 | 2.868 | .080 | 2.711 | 3.025 |

^a^Covariates appearing in the model are evaluated at the following values: AGE_D = 5.1087, IM=4.202, IA = 1.9292, HY_IM1 = 1.8517, HY_IM2 = 2.7355

The abbreviations for breed categories are: Toy – toy breeds; sTerr – small terriers; Basal – basal breeds; Herd – herding dogs; Mast – mastiffs; Retr – retrievers; Sight – sighthounds; Scent – scent dogs; Guard – guard dogs; Purebr – ungrouped purebreds; Cross – cross-breeds; Mixed – mixed breeds. For the basis of categorization see the *Methods*. For definition of Toy/object attachment levels see Supplementary Table S5.

**Text S3 Description of the laboratory reward responsiveness paradigm**

The dog is free to explore the test room (6.4m x 5.2m), where the spontaneous behaviour is measured in the passive presence of the owner and the experimenter.

*Objects, equipment used*

- 10 objects (around the size of an average shoe box) scattered in a random but fixed pattern on the floor: a plastic dvd case, an empty white plastic container, a rectangular wooden object with two barrels, a cylindrical plastic object with a grip, a closed plastic container, a big plastic spoon, an empty shoe box (paper), an empty metal box, an empty metal food can with two grips, an empty plastic bag
- a metal cage (size: 52x37x46 cm) with one side open (Episode 1, attainable reward) or closed (Episode 2, unattainable reward) placed in the middle of the room – only present during Episodes 1 and 2 (E1, E2)
- a plastic plate inside the open (E1) or closed (E2) cage
- a reward placed on the plastic plate, which is either a piece of sausage or cheese (Food responsiveness paradigm) or the dog’s favourite ball/toy (Ball/toy responsiveness paradigm)

*Familiarization phase (2 min)*

The dog, the owner and the experimenter enter the room, the dog is on leash and they stop with the owner in the doorway. The dog is immediately released to explore, while the owner and the experimenter stand close to the door and are chatting/talking to each other naturally, reacting naturally to the dog but not interacting with it physically. After 2 minutes the dog and the owner leave the room for approx. 30 sec.

*Episode 1 (2 min)*

Episode 1 always immediately follows the Familiarization phase, the only deviation from which is that a small piece of food (Food responsiveness paradigm) or a ball/toy (Ball/toy responsiveness paradigm) is available – the dog is free to eat/grab it – in the middle of the room in the open cage with the open side towards the door where the dog enters the room. The procedure is the same as described above in the Familiarization phase. If the dog does not notice the food/toy within 30 seconds, the owner attracts its attention to it (verbally and approaching and pointing closely towards the cage). After 2 minutes the dog and the owner leave the room for approx. 30 sec.

*Episode 2 (2 min)*

Episode 2 always immediately follows Episode 1. Everything is the same as described in Episode 1, except for the fact that the cage is closed so that the food/toy is unreachable for the dog, and the owner does not attract the dog’s attention to the reward after 30 sec if the dog does not show any interest towards it.

**Table S4 Definitions of variables measured in the laboratory reward responsiveness paradigm**

| **Behavioural paradigm** | | **Variable name** | **Definition** |
| --- | --- | --- | --- |
| **Food responsiveness** | food  reward  attainable (E1) | Approach_ food | Latency to first approach and eat the food (*s*) – measured from the moment the dog is off leash till the dog touches the food. |
|  |  | Near_ apparatus^*^ | Time spent near the cage (*%*) – the dog stays within approx. 15 cm range of the cage, regardless of orientation. |
|  |  | Manipulate_ apparatus^*^ | Time spent manipulating the cage (*%*) – the dog is physically manipulating (e.g. sniffing, pawing) the cage. |
|  |  | Back_ apparatus | Return frequency to near the cage (*n*) – calculated from the first approach |
|  | food  reward  unattainable (E2) | Approach_ apparatus | Latency to first approach the cage (*s*) – time passing from the moment the dog is off leash till the dog is in direct reach of the cage and focuses on the reward |
|  |  | Focus_food^*^ | Time spent focusing on the unattainable reward (*%*) – the dog gazes at the reward. |
|  |  | Manipulate_ apparatus^*^ | Time spent manipulating the cage (*%*) – the dog is physically manipulating (e.g. sniffing, pawing) the cage. |
|  |  | Near_ apparatus^*^ | Same as described above |
|  |  | Back_ apparatus | Return frequency to near the cage (*n*) - calculated from the first approach |
| **Ball/toy responsiveness** | ball/toy reward  attainable (E1) | Approach_ ball/toy | Latency to first approach and touch the ball/toy (*s*) - measured from the moment the dog is off leash till the dogs’ nose touches the ball/toy. |
|  |  | Near_ball/toy^*^ | Time spent near the ball/toy (*%*) – the dog stays within approx. 15 cm range of the ball/toy, regardless of orientation. |
|  |  | Manipulate_ ball/toy^*^ | Time spent manipulating the ball/toy (*%*) – the dog is physically manipulating (e.g. sniffing, chewing) the ball/toy. |
|  |  | Back_ball/toy | Return frequency to near the ball/toy (*n*) – calculated from the first approach |
|  | ball/toy  reward  unattainable (E2) | Approach_ apparatus | same as described above |
|  |  | Focus_ball/toy^*^ | same as described above by ‘Focus_food’ |
|  |  | Manipulate_ apparatus^*^ | same as described above |
|  |  | Near_ apparatus^*^ | same as described above |
|  |  | Back_ apparatus | same as described above |

^*^Measured from the first approach and calculated as % of total time after the first approach till the end of the episode.

**Table S5 Explanation of variables and corresponding factor levels included in the GLM and/or MANOVA**

| **Variable name** | **Description** | **Levels and definitions** | **Value** |
| --- | --- | --- | --- |
| Training method (TM) | The rewarding method used during the dog’s training; factor with 3 levels | food reward only | 1 |
|  |  | food and ball/toy reward | 2 |
|  |  | ball/toy or social reward only | 3 |
| Toy/object attachment (T/OA) | The dog’s level of attachment to its favourite object; factor with 4 levels | no attachment (*“does not have a favourite object”* or *“is not really attached to it”)* | 1 |
|  |  | only playing – *“likes to play with it, likes it to be near him/her but does not look for it when it’s not there”* | 2 |
|  |  | weak attachment – “*notices if it’s not there, but looks for it only for a short time”* | 3 |
|  |  | strong attachment – *“is restless if it’s not there, looks for it till he/she finds it”* or *“is badly attached to it, it can hardly be taken away from him/her”* | 4 |
| Reward frequency (RF) | The frequency with which the dog receives rewards (ball/toy or treats) for obedience tasks; factor with 4 levels | monthly | 1 |
|  |  | weakly | 2 |
|  |  | once daily | 3 |
|  |  | more times a day | 4 |
| Increase in motivation (IM) | 1-5 Likert scale variable;  *“It became more and more easy to motivate my dog with reward during the course of the training.”* | *“disagree completely”* | 1 |
|  |  | *“disagree moderately”* | 2 |
|  |  | *“partly agree, partly disagree”* | 3 |
|  |  | *“agree moderately”* | 4 |
|  |  | *“agree completely”* | 5 |
| Health problems due to excessive eating | 1-5 Likert scale variable;  *“My dog had physical problems as a consequence of his/her excessive eating (e.g. vomiting, diarrhoea, obesity, etc.)”.* | *“disagree completely”* | 1 |
|  |  | *“disagree moderately”* | 2 |
|  |  | *“partly agree, partly disagree”* | 3 |
|  |  | *“agree moderately”* | 4 |
|  |  | *“agree completely”* | 5 |
| Health problems due to excessive playing with ball/toys | 1-5 Likert scale variable; *“My dog had physical problems as a consequence of his/her excessive ball playing (e.g. accident, injury etc.)”.* | *“disagree completely”* | 1 |
|  |  | *“disagree moderately”* | 2 |
|  |  | *“partly agree, partly disagree”* | 3 |
|  |  | *“agree moderately”* | 4 |
|  |  | *“agree completely”* | 5 |
| Social problems due to excessive eating | Tow merged Likert scale variables*; “It annoys me that my dog likes to eat more than it should.”* and *“Sometimes I am angry with my dog because of his/her eating ’habits’.”* | Continuous scale (mean value of the two merged 1-5 Likert scale variables) | 1-5 |
| Social problems due to excessive playing with ball/toys | Tow merged Likert scale variables*; “It annoys me that my dog likes play ball/fetch objects frequently.”* and *“Sometimes I am angry with my dog because of his/her ball playing/object fetching ’habits’.”* | Continuous scale (mean value of the two merged 1-5 Likert scale variables) | 1-5 |

1. * Part of final CRRS items [↑](#footnote-ref-1)
2. * Part of final CRRS items [↑](#footnote-ref-2)
3. ** Multiple options possible [↑](#footnote-ref-3)
